# A novel chimeric vaccine containing multiple epitopes for simulating robust immune activation against *Klebsiella pneumoniae*

**DOI:** 10.1186/s12865-024-00617-z

**Published:** 2024-05-05

**Authors:** Morteza Hakimian, Abbas Doosti, Ali Sharifzadeh

**Affiliations:** 1 Department of Biology, Faculty of Basic Sciences, Shahrekord Branch, Islamic Azad University, Shahrekord, Iran; 2 Biotechnology Research Center, Shahrekord Branch, Islamic Azad University, Shahrekord, Iran; 3 Department of Microbiology, Faculty of Veterinary Medicine, Shahrekord Branch, Islamic Azad University, Shahrekord, Iran

**Keywords:** *Klebsiella pneumoniaee*, Reverse vaccinology, In silico analysis, Multi-epitopes chimeric vaccine

## Abstract

**Background:**

Due to antibiotic resistance, the *Klebsiella* genus is linked to morbidity and death, necessitating the development of a universally protective vaccine against *Klebsiella* pathogens.

**Methods:**

Core sequence analysis prioritized non-redundant host molecules and expected lipid bilayer peptides from fully sequenced *Klebsiella* genomes. These proteins were refined to identify epitopes, examining their immunogenicity, toxicity, solubility, and interaction with MHC alleles. Epitopes were linked to CPG ODN C274 via EAAAK, HEYGAEALERAG, and GGGS linkers to enhance immunological responses. The vaccine’s tertiary structure was modelled and docked with MHC-I and MHC-II.

**Results:**

Fifty-five proteins were recognized in the Vaxign collection as having remarkable features. Twenty-three proteins with potential pathogenicity were then identified. Eight options for vaccines emerged after the immunogenicity of proteins was examined. The best antigens were three proteins: MrkD, Iron-regulated lipid membrane polypeptides, and RmpA. These compounds were selected for their sensitivity. The structural protein sequences of *K. pneumoniae* were utilized to identify seven CTL epitopes, seven HTL epitopes, and seven LBL epitopes, respectively. The produced immunization displayed a stable contact with the receptors, based on molecular dynamic simulations lasting 250 nanoseconds. Intermolecular binding free energies also indicated the dominance of the van der Waals and electrostatic energies.

**Conclusion:**

In summary, the results of this study might help scientists develop a novel vaccine to prevent *K. pneumoniae* infections.

**Supplementary Information:**

The online version contains supplementary material available at 10.1186/s12865-024-00617-z.

## Background

The issue of antibiotic resistance poses a significant challenge to world health. Antimicrobial resistance (AMR) refers to the emergence of antibiotic resistance in microorganisms [1]. The emergence of antibiotic resistance might occur as a result of the continuous abuse of antibiotics [2]. The level of opposition is increasing significantly across many regions. The proliferation of new resistance mechanisms worldwide has led to a diminishing success rate in treating common viral illnesses. As a result, managing diseases resulting from bacterial infections has become more intricate and sometimes unattainable [3]. The Centers for Disease Control and Prevention (CDC) reports that illnesses caused by bacteria that are resistant to antibiotics lead to an annual incidence of 2.8 million cases and a mortality rate exceeding 35,000 [4].

The isolated clinical strains of Klebsiella pneumoniae are a notable contributor to nosocomial infections due to their extensive antibiotic resistance [5]. Moreover, Klebsiella pneumoniae is accountable for infections obtained within the community, particularly among those who have weakened immune systems or pre-existing medical disorders. K. pneumoniae infections have limited treatment options due to the development of resistance to β-lactam antibiotics [6]. In addition, Klebsiella pneumoniae, a natural producer of β lactamases, particularly extended-spectrum β-lactamases (ESBLs), has been shown to contribute to the development of antibiotic resistance [7]. Various approaches, such as botanical extracts and pharmaceuticals, have been used to address Klebsiella pneumoniae infection, yet the majority of these strategies have been ineffective. Klebsiella pneumoniae exhibits resistance to beta-lactam antibiotics. Consequently, this infection needs innovative treatment strategies [7].

Vaccination is an effective method to combat K. pneumoniae infections, contributing to antibiotic resistance development. Despite current studies on K. pneumoniae vaccines, none have received official approval [8]. Hence, in order to address this matter of public health, expeditious intervention is imperative. Despite their limited immunogenicity, pure polysaccharide vaccines have shown notable efficacy in providing 80% protection against isolates of K. pneumoniae, as evidenced by the findings of a study on a 24-valent capsular polysaccharide (SPC) vaccine [9]. The efficacy of vaccinations in mitigating antibiotic-resistant bacterial infections, such as K. pneumoniae, has been well acknowledged. However, their significance in managing these diseases has traditionally been underestimated [10].

Consequently, the development of vaccinations via technological progress will provide a viable resolution to the issue of antibiotic resistance. The development of vaccines has seen significant advancements as a result of progress in computer technology and genetic data. The field of reverse vaccinology (RV) employs a genome-based methodology to facilitate the creation of novel vaccine candidates. Recent studies have shown the beneficial use of RV, highlighting the need for vaccination as a preventive and cost-effective strategy against this virus. Researchers used reverse vaccinology and subtractive proteomics techniques in this study to ascertain prospective therapeutic interventions for A. baumannii [11, 16, 43]. The recombinant chimeric peptide vaccine demonstrated a 50% efficacy in hamsters against the pathogen during recent in vitro testing [12]. In addition, pan-genome-based RV (PGRV) development has been undertaken in response to the genomic variety seen in bacterial pathogens [13]. This approach aims to identify significant proteome antigens.

The efficacy of a subunit vaccine, which comprises an adjuvant, linker, and epitope, has been shown. Bioinformatics is of utmost importance in developing vaccines since it involves identifying and predicting key components, including the target antigen, B and T epitopes, and a suitable linker. Protein antigenicity plays a crucial role in shaping the design of vaccines. The accurate prediction of the subcellular localization of proteins is of utmost importance due to the immune system’s ability to promptly detect proteins present on the surface of pathogens. Klebsiella’s most notable surface proteins are MrkD, Iron-regulated outer membrane proteins, RmpA, YaiO, EMG21, TonB, ompA, ydiY, and OMVs. The immunogenicity of these proteins is attributed to their robust interaction with the immune system, which is facilitated by their positioning on the outer membrane [14–17].

Other approaches, like pang-genomics, immunoinformatic, and biophysical studies, may be used with the RV methodology to create a distinct multi-epitope polypeptide [14, 15]. After prioritizing different epitopes, a pan-proteome screening approach was used to ascertain the essential vaccination proteins [16]. Furthermore, the host immune system was guaranteed to accurately identify and handle the chosen epitopes, which are crucial for the pathogen’s survival and do not have any similar structures in humans. In order to ascertain immune responses specific to pathogens, conserved epitopes that have been shown to induce distinct B-cell responses were chosen.

## Methods

### Retrieval and analysis of protein sequences

The National Center for Biotechnology Information (NCBI) website (https://www.ncbi.nlm.nih.gov/) provided the *Klebsiella pneumoniae*. The selected strain’s genus, genome, and proteome were further investigated utilizing the web proteomics program Vaxign (https://violinet.org/vaxign/). The UniProt collection (https://www.uniprot.org/) was used to retrieve the complete proteome of this particular bacteria. A FASTA file extract was created using the selected protein sequences. Next, the VaxiJen v2.0 webpage (http://www.ddgpharmfac.net/vaxijen/) was used to evaluate the immunogenicity of the antigens utilizing the standard threshold value. This server’s auto cross-covariance (ACC) conversion technique yields a 70–89% accurate prediction result. After that, the AllergenFP v1.0 service (https://ddgpharmfac.net/AllergenFP/) was used to determine the allergenic patterns of the selected antigens. With an accuracy of 88.9%, this server employs a novel descriptive adjective fingerprint method that is alignment-free. For determining the transmembrane (TM) helix, the TMHMM v2.0 platform—which depends on a hidden Markov model (HMM)—was used. Protein molecules with fewer TM helices have been selected for the following investigation stage because they are immunological and non-allergic.

### Forecasting the qualities of virulence, antigenicity, flexibility, and hydrophilicity

The pathogenicity characteristic of potential vaccine candidates was examined using the VirulentPred database (http://203.92.44.117/virulent/). The cascade SVM module with a threshold value of 0.5 (≥ 0.5) was used in this investigation. The database VaxiJen provides a reliable way to determine the antigenic characteristics of proteins. This server was used to describe the antigenic characteristics of proteins solely based on their physicochemical properties, as opposed to sequence alignment. The antigenicity of the chosen proteins was determined using the VaxiJen method (http://www.ddg-pharmfac.net/vaxijen/VaxiJen/VaxiJen.html), with a cut-off of 0.5. The median flexibility and hydrophilicity of proteins were also calculated using http://tools.immuneepitope.org. A protein’s degree of solubility may provide helpful information about how effectively it performs. Over thirty per cent of the molecules created in immunization efforts are insoluble. The SOLpro (https://protein-sol.manchester.ac.uk/) tool uses a two-step SVM technique to estimate protein solubility. As a result, SOLpro calculates the proteins’ solubility.

### Cytotoxic T lymphocyte (CTL) epitope identification and analysis

The cytotoxic T lymphocytes (CTLs) are critical components of the host defence system. Direct contact between these cells and infectious germs inside the body’s immune response may lead to their death. The NetCTL v1.2 webpage (http://www.cbs.dtu.dk/services/NetCTL/) was utilized to run the selected protein sequences to anticipate the CTL epitopes with high combination scores. To maintain the sensitivity and specificity levels at 0.74 and 0.98, respectively, a threshold level of 0.90 was used. The MHC-I binding program available on the IEDB webpage (http://tools.iedb.org/mhci/) was used to identify the MHC-I binding variants of each CTL epitope following the CONSENSUS approach. It was believed that CTL epitopes might be lowered if their percentile rank was equal to or less than 2 because a lower percentile rank indicates a stronger affinity. Subsequently, the VaxiJen v2.0 web page (http://www.ddg-pharmfac.net/vaxijen/), the AllerTOP v2.0 programs (https://www.ddg-pharmfac.net/AllerTOP/), the ToxinPred applications (http://crdd.osdd.net/raghava/toxinpred/), and the IEDB Class I Immunogenicity system (http://tools.iedb.org/immunogenicity/) were used to determine antigenicity, the allergenic arrangement, toxic effects assessment, and immunogenicity for each CTL epitope, respectively.

Using fivefold cross-validation, the AllerTop v2.0 program uses k-nearest neighbor (kNN) methods, amino acid identities, and ACC modification techniques to identify non-allergens within allergens with an 85.3% predicted performance. The ToxinPred service evaluates the properties of different proteins using support vector machines (SVM), a computational method, and a statistical toxicity prediction model. Immunogenicity modelling aimed to ascertain if an epitope would elicit an immune response. While developing vaccines, CTL immunostimulatory, non-toxic, non-allergenic, and antigenic epitopes with high C-scores are considered.

#### Helper T-Lymphocyte (HTL) epitope identification and evaluation

Helper T lymphocytes (HTLs) are a component of the immune system’s adaptive response that may recognize foreign antigens and activate B-cells and CTLs to destroy harmful microbes. Among the selected sequence data, 15-mer HTL epitopes were found using the MHC-II binding program of the IEDB collection (http://tools.iedb.org/mhcii/). We used the CONSENSUS method to determine the corresponding interaction alleles of the compounds while keeping consistency in mind, with a percentile rank cutoff of equal to or less than 2. For every HTL epitope, allergenicity, antigenicity, and toxicity were predicted using the AllerTOP v2.0 web page, VaxiJen v2.0 program, and ToxinPred webpage.

The ability to trigger cytokines was used to accurately evaluate antigenic, non-toxic, and allergic HTL epitopes. IFN-γ activates natural killer cells and macrophages, which may trigger specialized and native immune reactions. It also enhances MHC responsiveness to ligands and plays a critical role in inhibiting the growth of microorganisms. We utilized the 81.39% efficient IFN-epitope website (http://crdd.osdd.net/raghava/ifnepitope/) to estimate interferon-gamma (IFN-γ). Furthermore, the websites IL4pred (https://webs.iiitd.edu.in/raghava/il4pred/) and IL10pred (https://webs.iiitd.edu.in/raghava/il10pred/) were used to evaluate the inducing qualities of interleukin-4 (IL-4) and IL-10 (IL-10), respectively. For both servers, the threshold values were − 0.3. Both procedures were executed using SVM-based methods. The IL4pred and IL10pred technologies give 75.76 and 81.24% accuracy, respectively. We considered the generational capability of all three cytokines while selecting the HTL epitopes for the vaccination. We prioritized IFN induction and the IL4- or IL10-inducing qualities of the HTL epitopes for antigens where no epitope exhibited all three cytokine-inducing capacities.

### Linear B-lymphocyte (LBL) epitope identification and evaluation

B-cell epitopes are the primary agents that cause humoral or particular antibody responses. Using the ABCpred website (https://webs.iiitd.edu.in/raghava/abcpred/), the LBL epitopes among the selected sequence information were predicted. The web servers VaxiJen v2.0 (http://www.ddg-pharmfac.net/vaxijen/), AllerTOP v2.0 (https://www.ddg-pharmfac.net/AllerTOP/), and ToxinPred v2.0 (http://crdd.osdd.net/raghava/) were used to evaluate the antigenic, allergic, and toxic patterns of the predicted LBL epitopes. In addition, the iBCE-EL platform’s default parameters (http://www.thegleelab.org/iBCE-EL/) were used to estimate the probability scores of LBLs.

### Epitope conservation and determining human similarity

The shortlisted MHC-I and MHC-II epitopes were examined using the IEDB tools. The similarity of the epitope to the human proteome was evaluated to identify which epitope is homologous to the protein database. Since homologous epitopes would not be able to elicit an immune system response, only non-homologous epitopes were used. The human homology was ascertained using the protein BLAST module of the BLAST database (https://blast.ncbi.nlm.nih.gov/Blast.cgi). This research compared Homo sapiens (taxid: 9606) against other species using default values, with a threshold e-value of 0.05. Epitopes were categorized as non-homologous peptides when no hits were found below the threshold e-value.

### Docking investigations and peptide modelling

CTL epitopes were docked with the HLA interacting allele to evaluate the identified CTL epitopes’ ability to bind. The PEP-FOLD v3.5 webpages (https://bioserv.rpbs.univ-paris-diderot.fr/services/PEPFOLD3/) include 200 simulations applied using the sOPEP sorting approach for the respective CTL epitopes. The sOPEP sorting service uses a de novo approach to determine the structural changes of small peptides (5–50 amino acids). Conformational changes are predicted using the Taboo/Backtrack sampling approach. This service was used to determine the probable structure of each peptide sequence. The efficiency of each structure was assessed using SWISS-PDB VIEWER, and the complex with the lowest energy was selected for further analysis. The co-crystallized ligand epitopes were selected as a control sample for the docking techniques, and human HLA-A*03:01 (PDB ID: 6O9B), HLA-A*01:01 (PDB ID: 6AT9), HLA-B*35:01 (PDB ID: 1A1N), and HLA-B*14:02 (PDB ID: 3BXN) variations were selected as CTL epitopes. The co-crystallized receptor version’s HLA allele composition was obtained from the RCSB Protein Data Library (https://www.rcsb.org/). In order to create proteins, ligands had to be removed from the molecules and hydrogens and Gasteiger-Marsili charges had to be added. UCSF Chimera’s protein synthesis wizard (version 1.11.2) was used to insert hydrogen. Next, Open-Babel was used to convert this file into the pdbqt format. The ligand energy form was optimized and transferred to the pdbqt version using PyRx 0.8’s OpenBabel package. The docking parameters were left at standard in the present operation. The grid box dimensions for HLA-A*01:01, HLAB* 35:01, HLA-A*03:01, and HLA-B*14:02 were set to 60.64 × 73.76 × 45.49 Å, 62.33 × 45.41 × 70.54 Å, 73.20 × 52.65 × 53.24 Å, and 61.25 × 48.69 × 72.95 Å for the X, Y, and Z axes of AutoDock Vina. Lower docking scores indicate a stronger binding affinity. The molecular docking results are expressed as negative quantities in kcal mol − 1. The graphics were created using Microsoft PowerPoint 2019 and UCSF Chimera, and the molecular dynamics results were shown using Discovery Studio (DS) version 4.5.

### MHC clustering algorithm

The MHC genomic region shows widespread variation in most mammals. The human MHC genetic code (HLA) is highly variable, with hundreds of alleles. Most of the time, how specifically the MHC allele may work still needs to be determined. MHC allele clustering makes it possible to distinguish between the two types of MHC molecules by comparing their binding-specific traits. Using phylogenetic trees and default settings, heatmaps showing the functional clustering between MHC variants were produced using the MHC-cluster 2.0 webpage (http://www.cbs.dtu.dk/services/MHCcluster/). The HLA-prevalent and -characterized component was used to construct the NetMHCpan-2.8 approach for MHC class I cluster assessment. In contrast, the pertinent DRB allele elements were used for MHC class II clustering techniques.

### Construction of the vaccine concept

A vaccine was developed using the selected proteins’ particular LBL, CTL, and HTL epitopes. We connected the selected HTL, CTL, and LBL using HEYGAEALERAG and Gly-Gly-Gly-Ser (GGGS) linkers. The GGGS linker improves immunogenicity and epitope presentation, affecting binding affinity and protein stability. The HEYGAEALERAG linker promotes immunological processing and inhibits the formation of “junctional epitopes.” The HEYGAEALERAG linker was inserted to act as an interface between CTL and HTL epitopes.

### Assessment of the physicochemical, antigenicity, allergenicity, and solubility profiles

Using the ProtParam website (https://web.expasy.org/protparam/), the physicochemical properties of the vaccine construct were examined. These properties included the molecular weight (MW), conceptual isoelectric point (pI), aliphatic index (AI), instability index (II), half-life (both In vitro and In vivo), and grand average hydropathicity (GRAVY). Various techniques were used to investigate the vaccine’s immunologic, allergic, and hydrophilic properties. The ANTIGENPro application from the Scratch protein prediction site and the Vaxijen v2.0 website (accessible at http://www.ddg-pharmfac.net/vaxijen/) were also used to predict antigenicity. Cross-validation studies on the pooled dataset showed that ANTIGENPro was 76% accurate. AllerTop v2.0, AllergenFP v1.0, and AlgPred websites (http://crdd.osdd.net/raghava/algpred/) were used to simulate the allergenic characteristics of the vaccine design in light of the expectation of no allergic reaction. The solubility of the vaccine design was assessed using the Scratch protein prediction service’s SOLpro online software (http://scratch.proteomics.ics.uci.edu/). The vaccination is presumed to be soluble if the prediction value is 0.5 or above. Utilizing the Protein-Sol website (https://protein-sol.manchester.ac.uk/) also improved my understanding of solubility. The protein is soluble if the projected value is more than 0.45. The number of transmembrane helices was predicted using the TMHMM v2.0 webpage (http://www.cbs.dtu.dk/services/TMHMM/). Verify 3D and ERRAT were two validation tools used to assess the tertiary structure’s quality. RMSD was employed as a quantitative metric to assess the similarity between protein structures, density, temperature, and total energy plots.

### In-silico cloning and codon optimization of a proposed vaccine

Wrangler (https://www.mrc-lmb.cam.ac.uk/ms/methods/codon.html) and JCAT (http://www.jcat.de/) web server applications were used to optimize heterologous protein production in the mouse model. The RF-Cloning technology (https://rf-cloning.org/) was used to guarantee vaccine expression, and the final vaccine constructs DNA sequence was created in vector pcDNA3.1(+). Numerous enzyme restriction sites, autonomous transcription terminators, and prokaryotic ribosome receptors were also created.

## Results

### A potential vaccine candidate has been recognized

**Fig. 1:**
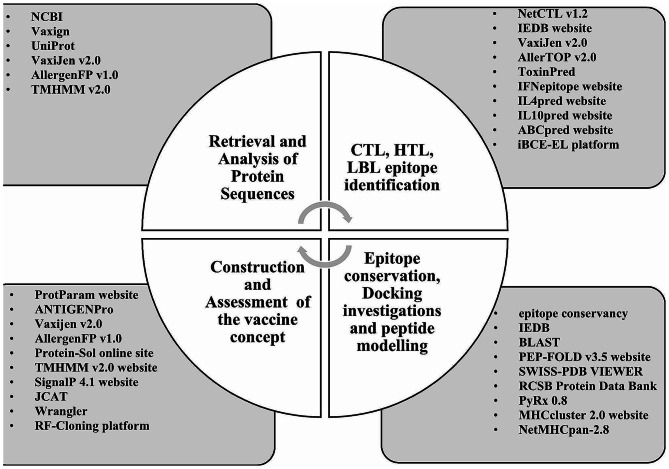
Genome assembly and annotation report from 86 *Klebsiella pneumoniae* strains

For this investigation, 68 completely sequenced *Klebsiella pneumoniae* genomes were obtained from the NCBI. Complete proteome information for the strain may be found at https://www.ncbi.nlm.nih.gov/genome/?term=Klebsiella+pneumoniae. The genomes of *Klebsiella pneumoniae* utilized are available at the following link: https://www.ncbi.nlm.nih.gov/genome/browse#!/prokaryotes/815/. Every genome encoded around 5.59628 megabytes of proteins. Each strain had a different quantity of proteins, but the average protein number was 5301. The number of genomes identified in each strain of *Klebsiella pneumoniae* is shown in Fig. 1. The core-pan plot indicates that, due to chromosomal flexibility, the pathogen’s expected proteome is open and that the probability of acquiring new genes over time is relatively high.

Moreover, examining COG patterns demonstrated that the core proteins mostly mediated the metabolic biogenesis. Data processing and storage were associated with the distinct set of proteins present in *Klebsiella pneumoniae* subsp. pneumoniae HS11286 (reference strain). Four categories may be used to categorize the data: recombination, transcription and translation, replication, and RNA processing (Table S1).

**Fig. 2:**
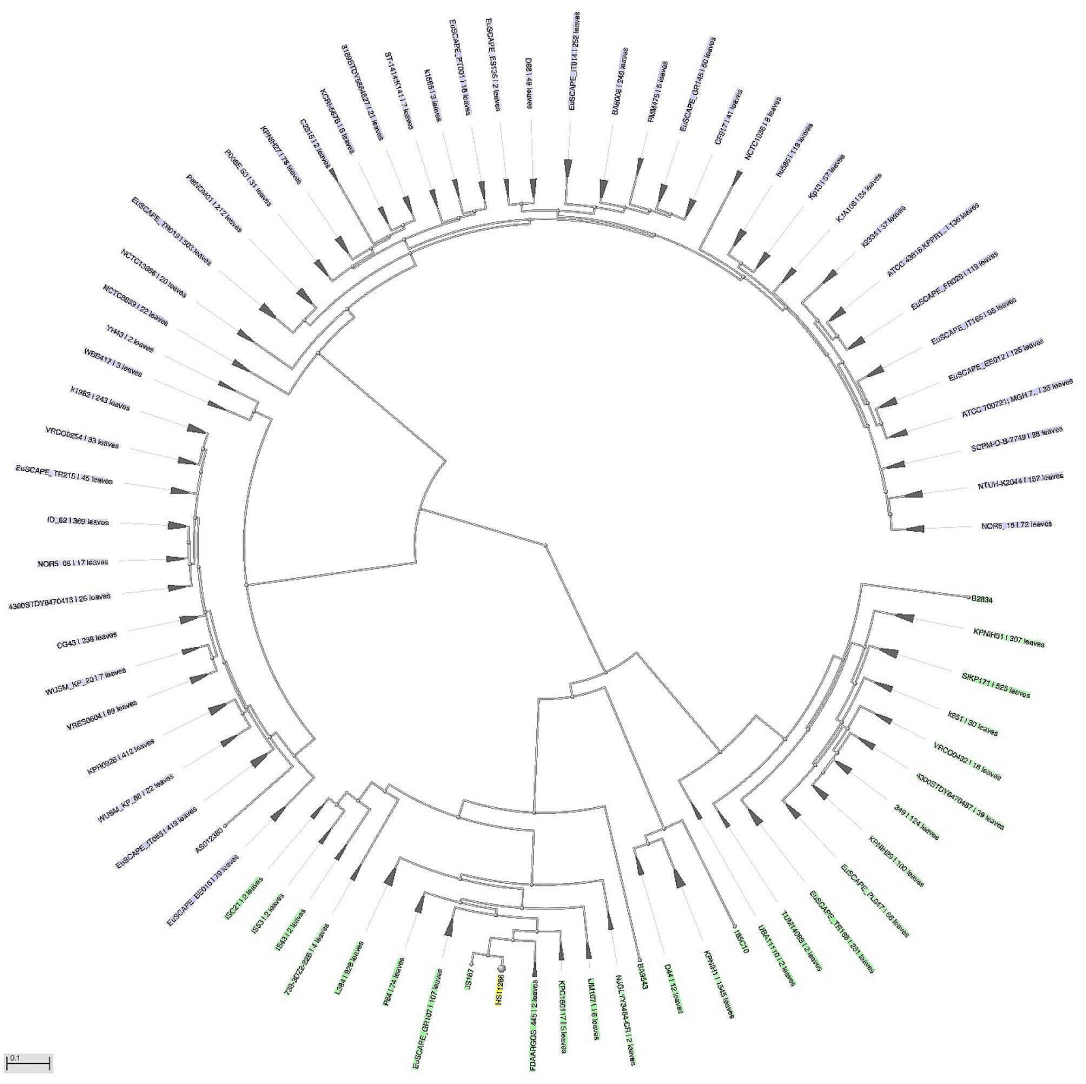
The reverse vaccinology technique was utilized to identify new prospective *Klebsiella pneumoniae* vaccine candidates

A dataset from Vaxign identified 55 proteins with remarkable characteristics. Twenty-three proteins with possible pathogenicity were then selected. Following an assessment of the antigenicity of proteins, eight possible vaccine candidates remained. Table 1 shows this technique in action. The accession number of the proteins was selected on the NCBI webpage after the Uniport databases confirmed the presence of eight different proteins in *K. pneumoniae*. They were classified as extracellular, outer membrane, periplasmic, inner membrane, and cytoplasmic protein types based on the results of the CELLO algorithm (Fig. 2). The specificity of the proteins chosen for *K. pneumoniae* was confirmed by BLASTp findings, reducing the likelihood that the immunization may interact with host cells. Table 1 displays the results of the first screening of eight *K. pneumoniae* proteins. Based on the preliminary screening findings, MrkD, Iron-regulated outer membrane proteins, and RmpA proteins were the most appropriate antigens chosen for immunogenicity (Table 1).

**Table 1:** Screening of *Klebsiella pneumoniae* proteins

Name	Accession Number	CELLO analysis		Protein- Sol		IEDB	VaxiJen
		Localization	Reliability	SOLpro	**PI**	Flexibility	
MrkD	AGZ83319.1	Extracellular	3.44	0.49	9.73	1.003	0.82
		OuterMembrane	1.49				
Iron-regulated outer membrane proteins	VVJ51509.1	Extracellular	4.46	0.40	4.86	1.005	0.58
		OuterMembrane	0.36				
RmpA	AUG89078.1	Periplasmic	1.27	0.59	10.13	0.987	0.46
		OuterMembrane	0.91				
YaiO	TNA22439.1	OuterMembrane	2.23	0.36	6.66	0.993	0.49
		Extracellular	2.42				
EMG21	TMZ64363.1	Extracellular	2.01	0.53	9.18	1.008	0.56
		OuterMembrane	1.43				
TonB	CAA48498.1	Periplasmic	3.63	0.75	10.09	1.012	0.76
		OuterMembrane	0.30				
ompA	CDO13836.1	OuterMembrane	4.50	0.39	6.24	1.000	0.64
		Periplasmic	0.32				
ydiY	CDO15043.1	OuterMembrane	2.23	0.58	5.02	1.004	0.69
		Extracellular	2.42				

### T lymphocyte epitopes chosen

Gene database searches led the PSORT II server to predict that three amino acid sequences (MrkD, Iron-regulated outer membrane proteins, and RmpA) were probably located in the plasma membrane. Through CCTOP prediction, we were able to identify 48 extracellular domains.

The promiscuous CTL epitopes for 50 alleles were predicted using the IEDB server. The list of HLA alleles used in this study to ascertain the MHC variant sequence is shown in Table S2. Three thousand two hundred twenty-one epitopes with a percentile rank of less than 0.5 for each extracellular domain were found. Based 18 alleles, IEDB predicted 2,107 HTL epitopes with percentile rank < 0.5 and IC50 < 500 nM. Different epitopes have been assigned to several MHC alleles and processed as a single entity in later research.

### Linear B-cell epitopes

For each of the 48 receptor complexes, linear B-cell epitopes were found using the ABCpred service. The investigation produced 985 sequences in all.

### The convergence of cellular and humoral stimulatory epitopes

The alignment of different sequences facilitates the discovery and choosing of epitopes with the capacity to induce humoral and cellular responses. After the first alignment between B-cell epitopes and CTLs, a list of CTL epitopes overlapped with B-cell patterns was produced. By using class I immunogenicity evaluation, another set of peptides was discovered. The best CTL epitopes were gathered as the final product. Similarly, a list of HTL epitopes overlapping with B-cell genomes was generated and refined by removing those whose IC50 was less than 50 nM. In order to offer 48 matching sequences, the best epitope categories (CTLs and HTLs) were finally aligned.

**Table 2:** Selected CTL and HTL epitopes overlapping with each other and with B-cell epitopes

Protein Code	CTL epitope	HTL epitope	B-Cell epitope	interacting MHC-I alleles	human homology
AGZ83319.1	---	---	ATVNIVYPDVFSSRVYNTTNYSLEGS	---	non-homologue
	CTRLS**SPTV**	**SPTV**MLDMVVGRVVV	S**SPTV**MLDM	HLA-A*24:02 HLA-DRB1*03:01	non-homologue
	**TMSAPGG**ASY	LTRDW**TMSAPGG**ASY	DW**TMSAPGG**	HLA-B*15:01, HLA-DRB1*09:01	non-homologue
	WESGGNPIL	YDIYVNMQGNVKNNI	FAAKIVSPGATDLGNKIYSTNV	HLA-B*40:01, HLA-DRB5*01:01	non-homologue
	GSVILTRDW	FMYLYSLNKEMVDER	ATVNIVYPDVFSSRVYNTTNYSLEGS	HLA-B*57:01, HLA-DRB5*01:01	non-homologue
	MSAPGGASY	KLYFAFLKKNVSRIV	ATTGSGTL	HLA-DRB3*02:02, HLA-A*01:01	non-homologue
	TMSAPGGASY	AFLKKNVSRIVNHYP	YDWESGGNPIL	HLA-B*15:01, HLA-DRB3*02:02	non-homologue
	MSLRKLLTLF	GGYKMLRGSLNMISQ	IRRTDLKGVGTTAGGK	HLA-DRB1*04:05, HLA-B*57:01	non-homologue
	GEVESHMIF	VKNNIEKLYFAFLKK	SSPTVMLDM	HLA-B*44:03, HLA-DPA1*01:03/DPB1*04:01	non-homologue
	RYITIPLHAR	KFGCKRYIRFMYLYS	DWTMSAPGG	HLA-DPA1*02:01/DPB1*05:01, HLA-A*31:01	non-homologue
	RLNNQETRY	RYIRFMYLYSLNKEM	GGLSETGYA	HLA-A*30:02, HLA-DPA1*01:03/DPB1*04:01	non-homologue
	YLSANAITV	FGCKRYIRFMYLYSL	ATSTTATMGALLNEKAGSGMA	HLA-A*02:03, HLA-DPA1*02:01/DPB1*05:01	non-homologue
	ITIPLHARFY	EKLYFAFLKKNVSRI	QFNKKYTVGRLNNQETRYI	HLA-B*57:01, HLA-DPA1*02:01/DPB1*05:01	non-homologue
VVJ51509.1	**EQQVQGGKE**F	Q**EIEQQVQGGKE**FKD	GQEI**EQQVQGGKE**	HLA-A*26:01, HLA-DRB1*12:01	non-homologue
	**QSDE**DSIIV	AA**QSDE**DSII VSAN	AA**QSDE**	HLA-B*15:01, HLA-DQA1*01:01/DQB1*05:01	non-homologue
	VSGGVRYQW	TLPALAAQSDEDSII	YYKSQGDDDYGLWLGKNMSAVTSGGKAYTTDGLNSDRIPGTE	HLA-B*57:01, HLA-DQA1*04:01/DQB1*04:02	non-homologue
	NTVDFIGSY	HPLLLASTLPALAAQ	YRDESLTFYPFPTLTKGQVSSFSSSQQDTDQ	HLA-A*26:01, HLA-DPA1*02:01/DPB1*14:01	non-homologue
	VYYRDESLTF	NANQMFFDLPQSMAS	HETFNANQMFFDLPQSMASGGLHNESIYTTGRYPGYS	HLA-A*23:01, HLA-DQA1*01:01/DQB1*05:01	non-homologue
	IEIDDNRQL	LVSQVYYRDESLTFY	ENRVDDFVGYAQQQDIANGKARSADAIKGGKTDYDNF	HLA-B*40:01, HLA-DRB3*01:01	non-homologue
	GSYALPVGK	DNLRTQLAAYYSTSD	GVELPDPGKYYGIGKYGAAVNGHLPLISSVNVDDSPLQ	HLA-A*11:01, HLA-DRB1*12:01	non-homologue
	QTDGRWQKW	GDNLRTQLAAYYSTS	VNRTDMTIDVQSDK	HLA-B*57:01, HLA-DRB1*12:01	non-homologue
	HPLLLASTL	LVSQVYYRDESLTFY	TDGRWQKWDVTLASP	HLA-DRB1*04:01, HLA-B*07:02	non-homologue
	ETFNANQMF	FFGQNLVSQVYYRDE	PDPWSLRVQSQQVFDLSDAAGNKLEG	HLA-A*26:01, HLA-DRB3*02:02	non-homologue
	LQGIKVNSY	GYSISNVAPFLQSSY	YVTIWGQRAPLLYSPTYGSSSLYEYKG	HLA-DRB1*09:01, HLA-B*15:01	non-homologue
AUG89078.1	**LTDDYFFYY**	NIV**LTDDYFFYY**GLK	---	HLA-A*01:01, HLA-DRB3*01:01	non-homologue
	**KLYFAFLKK**	E**KLYFAFLKK**NVSRI	---	HLA-A*03:01, HLA-DRB1*11:01	non-homologue
	**ITYEGVVNK**	FH**ITYEGVVNK**SIAI	**VVNK**SIAIK	HLA-A*11:01, HLA-A*03:01, HLA-DRB5*01:01	non-homologue
	GSLNMISQW	EKLYFAFLKKNVSRI	GGINEIKSQLKIEEKT	HLA-B*57:01, HLA-DRB1*11:01	non-homologue
	KSIAIKHKR	LYFAFLKKNVSRIVN	KEMVDERWLM	HLA-A*31:01, HLA-DRB1*11:01	non-homologue
	DVSGGGRFY	GGYKMLRGSLNMISQ	SGGGRFYPKGCDYDIYVNMQGNVKNN	HLA-A*26:01, HLA-DRB1*01:01	non-homologue
	KTLSCYQSK	FFYYGLKQLTGLPLF	SGKWGGYKMLRGSLNMIS	HLA-DPA1*02:01/DPB1*01:01	non-homologue
	LNMISQWMW	LQCLLKNGGINEIKS	SRIVNHYPRLTKK	HLA-B*58:01, HLA-DRB1*13:02	non-homologue

### Human homology and epitope conservation

Every chosen MHC class epitope showed 100% maximal identity for conservation. Additionally, selected CTL, HTL, and LBL epitopes have been demonstrated to be non-homologous to the human amino acid database, corroborating the epitopes’ classification as antigens or exogenous materials inside the human body. Table 2 presents studies of human homology and conservation for the selected epitopes.

### Allele and CTL epitope molecular docking analyses

It would help to search for a protein structure whose chemical makeup is comparable to that of the crystalline ligand to get reliable binding data. The alleles with suitable interactions with our receptor were chosen for the docking study. The proper allelic interactions were determined on the IEDB website. The binding impact of the chosen CTL epitopes with their respective HLA alleles was examined using molecular docking modelling. We selected the HLA-A*24:02 and HLA-DRB1*03:01 alleles for the CTRLSSPTV, SPTVMLDMVVGRVVV, and SSPTVMLDM epitopes of the MrkD protein. HLA-A*26:01 and HLA-DRB1*12:01 were selected for the EQQVQGGKEF, QEIEQQVQGGKEFKD, and GQEIEQQVQGGKE epitopes of iron-regulated outer membrane proteins. A*11:01, A*03:01, and HLA-DRB5*01:01 were selected for the RmpA protein’s ITYEGVVNK, FHITYEGVVNKSIAI, and VVNKSIAIK epitopes. Furthermore, as Fig. 1 illustrates, distinct alleles were selected for the epitopes of RmpA, MrkD, and Iron-regulated outer membrane proteins.

Crystallized ligand epitopes were used as the positive control to validate the docking experiments. Molecular docking results connected the HLA alleles, specific CTL epitopes, and positive controls. All positive controls had higher binding affinities than other specific CTL epitopes, except the HLA-A*24:02 control, where TMSAPGGASY (-10 kcal/mol) and LTRDWTMSAPGGASY (-9.2 kcal/mol) epitopes produced higher docking amounts than the positive control (-9.1 kcal/mol) of the HLA-A*24:02 allele. They possessed fewer hydrogen bond linkages than the positive control of HLA-A*24:02, even though their docking values were greater. The interactions induced among the specific alleles and the favorable controls were similar to those between the CTL epitopes and the particular ligands. With the HLA-A*26:01 alleles, EQQVQGGKEF produced the most significant number of hydrogen bonds—eight—while the control HLA-A*01:01 allele revealed the most significant number of hydrogen bonds—12. The positive control HLA-B*14:02 (-10.0 kcal/mol) exhibited higher docking scores than the epitopes LQGIKVNSY (-8.2 kcal/mol) and LNMISQWMW (-7.6 kcal/mol). However, these epitopes formed more hydrogen bonds than the control group. Furthermore, hydrophobic linkages allowed the CTL epitopes and positive controls to engage with the receptors.

### Evaluation of population coverage

**Fig. 3:**
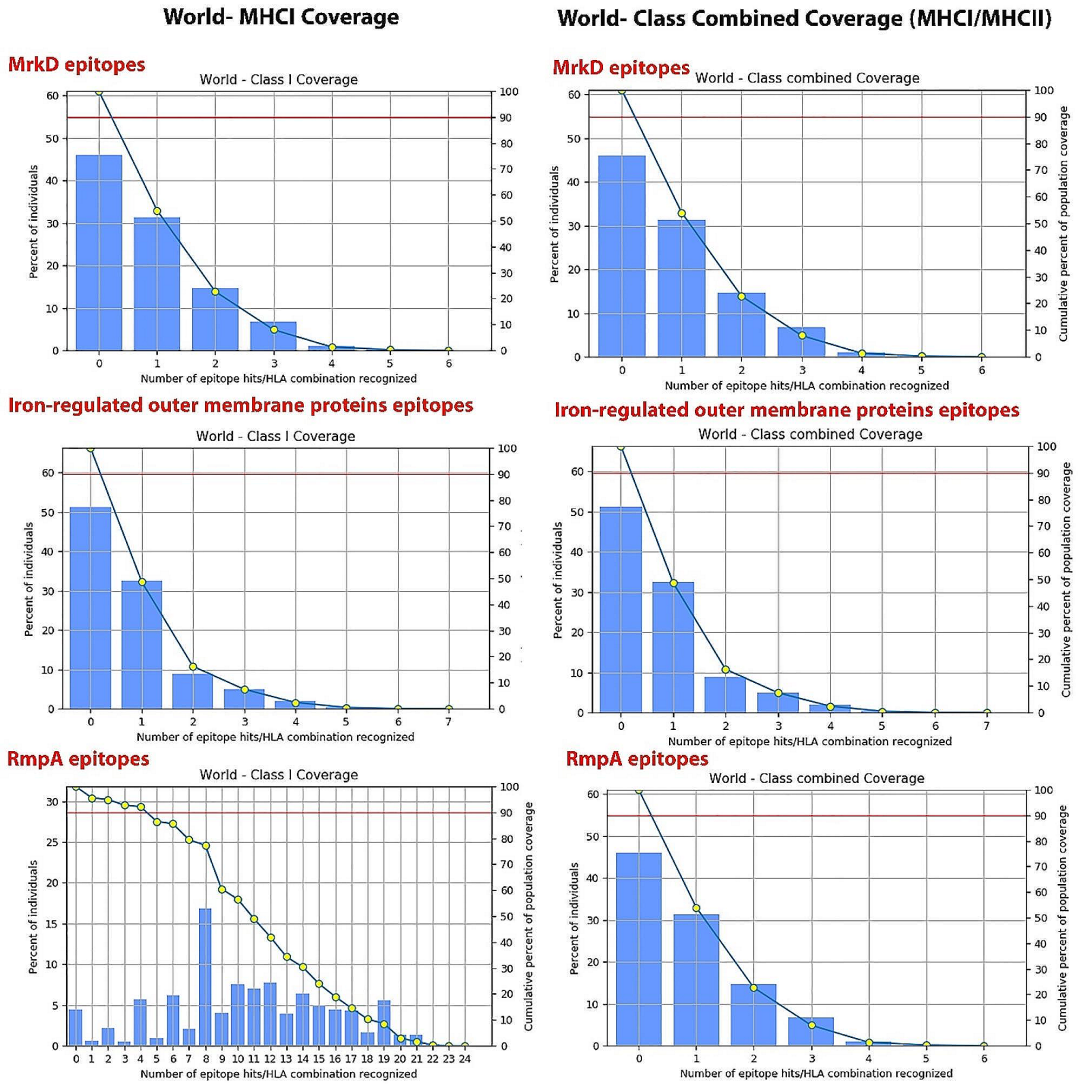
Population Coverage Calculation Result for MrkD, Iron-regulated outer membrane proteins and RampA epitopes. the results of the population coverage study for the RmpA protein MHCI and class combination epitopes, which showed 44.37% and 50.79% worldwide coverage, respectively

In the population coverage investigation, the global coverage of the MrkD antigen MHCI and class combination epitopes was 54.01% and 59.28%, respectively. According to the community prevalence study, the combined epitopes of the MHC class and the iron-regulated outer membrane proteins MHCI showed 53.74% and 48.66% worldwide coverage. Table S3, https://s24.picofile.com/file/8454030850/S3_TABLE.docx.html, shows the results of the population coverage study for the RmpA protein MHCI and class combination epitopes, which showed 44.37% and 50.79% worldwide coverage, respectively. We focused on the combined population coverage of both MHC epitopes in vaccine proteins. 79% of the world’s population was covered. The regions with the highest population coverage for combined MHC Class-I and class-II epitopes were Oceania (79.22%), North America (82.12%), West Africa (75.19%), Northeast Asia (81.21%), North Africa (86.05%), South Africa (74.56%), South Asia (64.38%), East Africa (52.98%), Southwest Asia (57.20%), West Indies (74.56%), Central Africa (81.09%), Southeast Asia (53.71%), East Asia (76.27%), South America (61.22%), and Central America (51.22%) (Fig. 3).

### MHC cluster evaluation results

**Fig. 4:**
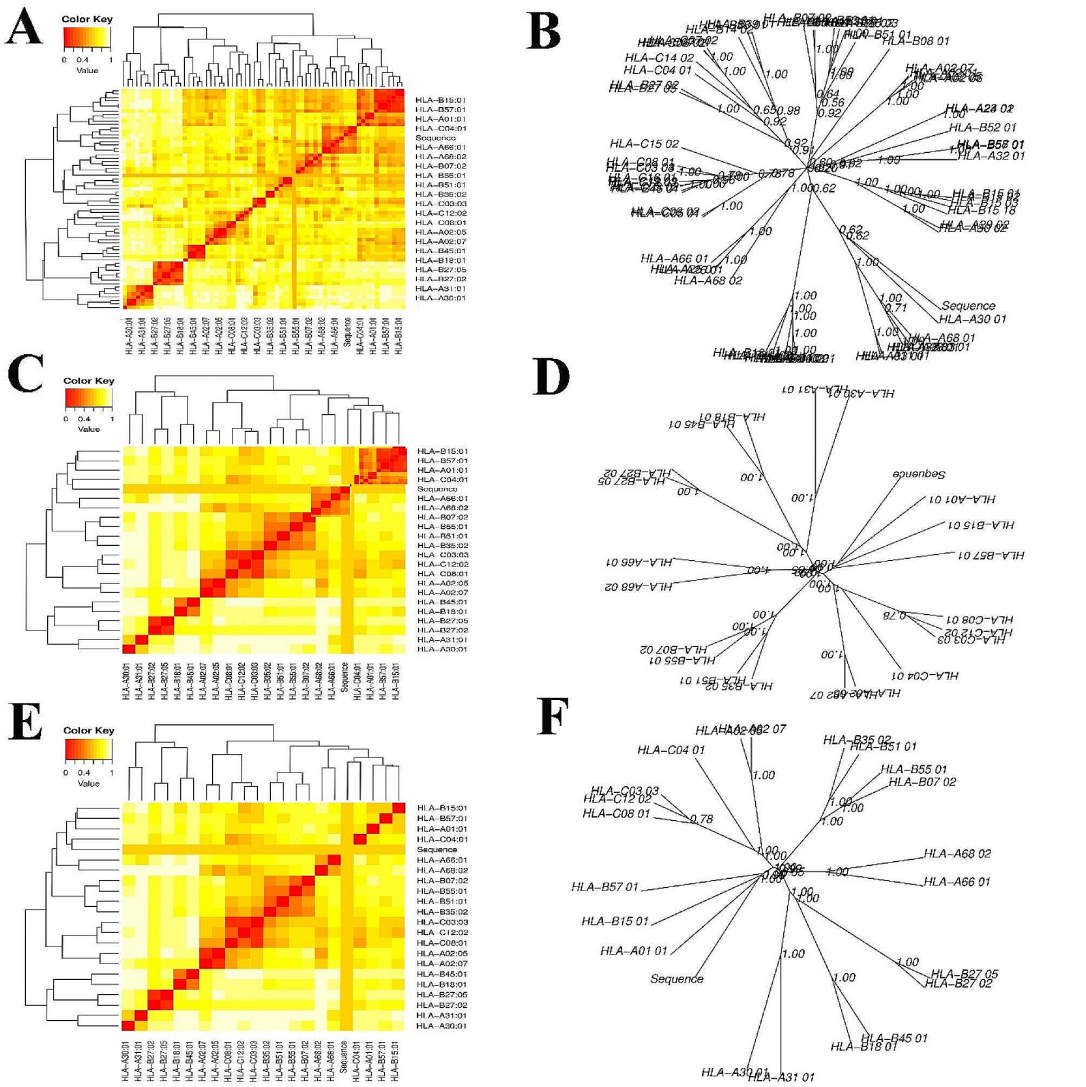
The findings of the MHC clustering algorithms. Heatmap of MHC class-I cluster assessment of (**A**) MrkD, (**C**) Iron-regulated outer membrane proteins, and (**E**) RmpA protein. Advanced tree map of MHC Class-I cluster evaluation of (**B**) MrkD, (**D**) Iron-regulated outer membrane proteins, and (**F**) RmpA protein. The read portions of the heatmap showed higher relationships between the clustered HLA alleles, while the heatmap’s yellow sections showed lesser connections between clustering HLA alleles

The MHCcluster v2.0 database categorized the MHCI and MHCII variants based on their interactions with the designated structural proteins’ epitopes. Twenty-one alleles from each MHC class are included in the study: HLA-B15:01, HLA-A02:05, HLA-A01:01, HLA-A02:07, HLA-A30:01, HLA-B27:02, HLA-A66:01, HLA-A68:02, HLA-B07:02, HLA-B18:01, HLA-B27:05, HLA-A31:01, HLA-B45:01, HLA-B51:01, HLA-B55:01, HLA-B57:01, HLA-C03:03, HLA-C04:01, HLA-C08:01, and HLA-C12:02 in each MHC class. Figure 4A and C illustrate the clustering pattern for the MHCI and MHCII alleles. An enhanced tree diagram of the MHCI and MHCII clustering results is shown in Fig. 4B, D and E, and 4F. The heatmap’s red areas revealed stronger linkages between the concentrated HLA alleles, while its yellow areas revealed weaker correlations.

### The Vaccine Construct is being developed

The most potent LBL, CTL, and HTL epitopes were combined with HEYGAEALERAG and GGGS linkers to create the vaccine design. The adjuvant CPG ODN C274 (5’-TCGTCGAACGTTCGAGATGAT-3’) was attached to the front of the vaccination using the EAAAK linker. A CpG ODN C274-mediated rat TLR-9 stimulation in NF-kB LeeporterTM—RAW 264.7 cells promote overall IgG antibodies, Th1 cell protection, and the TLR-9 pathway. From the structural protein sequences of *K. pneumoniae*, seven CTL, seven HTL, and seven LBL epitopes were selected for the present investigation, respectively. After meticulous combining and randomizing, we designed a vaccine. Table 3 displays the completed vaccination build.

**Table 3:** Sequence of vaccine structures designed in this study

Epitope composition	group	Vaccine sequence	AlgPred	SOLpro (< 0.4)	PI	GRAVY	VaxiJen	Homology
Multi epitope for KELEBSIELLA	Adjuvant + 7CTL + 7HTL + 7LBL	MSSNVRDDSSNVRDDSSNVRDDSSNVRDDSSNVRDDEAAAKCTRLSSPTVHEYGAEALERAGTMSAPGGASYGGGSEQQVQGGKEFGGGSQSDEDSIIVGGGSLTDDYFFYYGGGSKLYFAFLKKGGGSITYEGVVNKGGGSSPTVMLDMVVGRVVVGGGSLTRDWTMSAPGGASYGGGSQEIEQQVQGGKEFKDGGGSAAQSDEDSIIVSANGGGSNIVLTDDYFFYYGLKGGGSEKLYFAFLKKNVSRIGGGSFHITYEGVVNKSIAIGGGSATVNIVYPDVFSSRVYNTTNYSLEGSGGGSATVNIVYPDVFSSRVYNTTNYSLEGSGGGSYYKSQGDDDYGLWLGKNMSAVTSGGKAYTTDGLNSDRIPGTEGGGSHETFNANQMFFDLPQSMASGGLHNESIYTTGRYPGYSGGGSENRVDDFVGYAQQQDIANGKARSADAIKGGKTDYDNFGGGSVVNKSIAIKHEYGAEALERAGSGKWGGYKMLRGSLNMISEAAAK	NON ALLERGEN	0.516	4.99	-0.33	1.13	non-homologue
	Adjuvant + 7CTL + 7HTL + 7LBL + Adjuvant	MCRTFEMEAAAKCTRLSSPTVHEYGAEALERAGSPTVMLDMVVGRVVVGGGSATVNIVYPDVFSSRVYNTTNYSLEGSGGGSTMSAPGGASYGGGSLTRDWTMSAPGGASYGGGSATVNIVYPDVFSSRVYNTTNYSLEGSGGGSEQQVQGGKEFGGGSQEIEQQVQGGKEFKDGGGSYYKSQGDDDYGLWLGKNMSAVTSGGKAYTTDGLNSDRIPGTEGGGSQSDEDSIIVGGGSQEIEQQVQGGKEFKDGGGSHETFNANQMFFDLPQSMASGGLHNESIYTTGRYPGYSGGGSLTDDYFFYYGGGSNIVLTDDYFFYYGLKGGGSENRVDDFVGYAQQQDIANGKARSADAIKGGKTDYDNFGGGSKLYFAFLKKGGGSEKLYFAFLKKNVSRIGGGSVVNKSIAIKGGGSITYEGVVNKGGGSFHITYEGVVNKSIAIHEYGAEALERAGSGKWGGYKMLRGSLNMISEAAAKCRTFEM.	NON ALLERGEN	0.506	5.11	-0.38	1.13	non-homologue

### Antigenicity, allergenicity, and physicochemical profile of vaccines

The Expasy website assessed the physicochemical characteristics of the produced component. Vaccine-1’s molecular formula was found to be C2244H3428N622O764S16. The vaccine-2 form has 39 positively charged residues (Arg + Lys) and 49 negatively charged residues (Asp + Glu), with a molecular weight of 51856.57 Da, indicating a mean mass. Using the Expasy website, the chemical formula for vaccine-2 was C2262H3453N627O766S16. The vaccination-2 construct has a molecular weight of 52200.00 Da, 50 negatively charged residues (Asp + Glu), and 39 positively charged residues (Arg + Lys). For both vaccines, the predicted half-lives were 7.2 h (for mammalian reticulocytes In vitro), > 20 h (for yeast In vivo), and > 10 h (for *Escherichia coli* In vivo).

For vaccines 1 and 2, the expected PI characteristics of 4.99 and 5.11 indicated that the vaccination was alkaline, respectively. Vaccine-1 had an aliphatic index value of 57.04 and an instability value of 40.02. Vaccine-2’s aliphatic index value was 55.58, and the instability score was 41.05. The structure was hydrophilic (GRAVY) based on the grand mean of hydropathicity values (vaccine-1: -0.33, vaccine-2: -0.38). The vaccines’ antigenicity and allergenicity were developed. Using the Vaxijen2.0 server, the antigenicity values for vaccines-1 and vaccine-2 were found to be 1.13 and 1.1364, respectively. Interestingly, all servers demonstrated that the immunizations were non-allergic and antigenic. Additionally, the Protein-Sol server was soluble in the vaccine design, which gave vaccine-1 and vaccine-2 values of 0.516 and 0.506, respectively (Table 3). Moreover, the corresponding prediction results for the vaccine constructions did not include TM helices or signal peptides.

### Blast homology analysis

Table 3 shows that there was no similarity (0%) between the vaccine construct’s query coverage and the proteins of Homo sapiens, based on the sequence homology between the generated vaccination protein sequence and the human proteome sequence. The anticipated vaccine protein will not cause autoimmune responses in the host, according to the results of the BLAST homology study.

### Prediction of secondary and 3D structure of vaccines

**Fig. 5:**
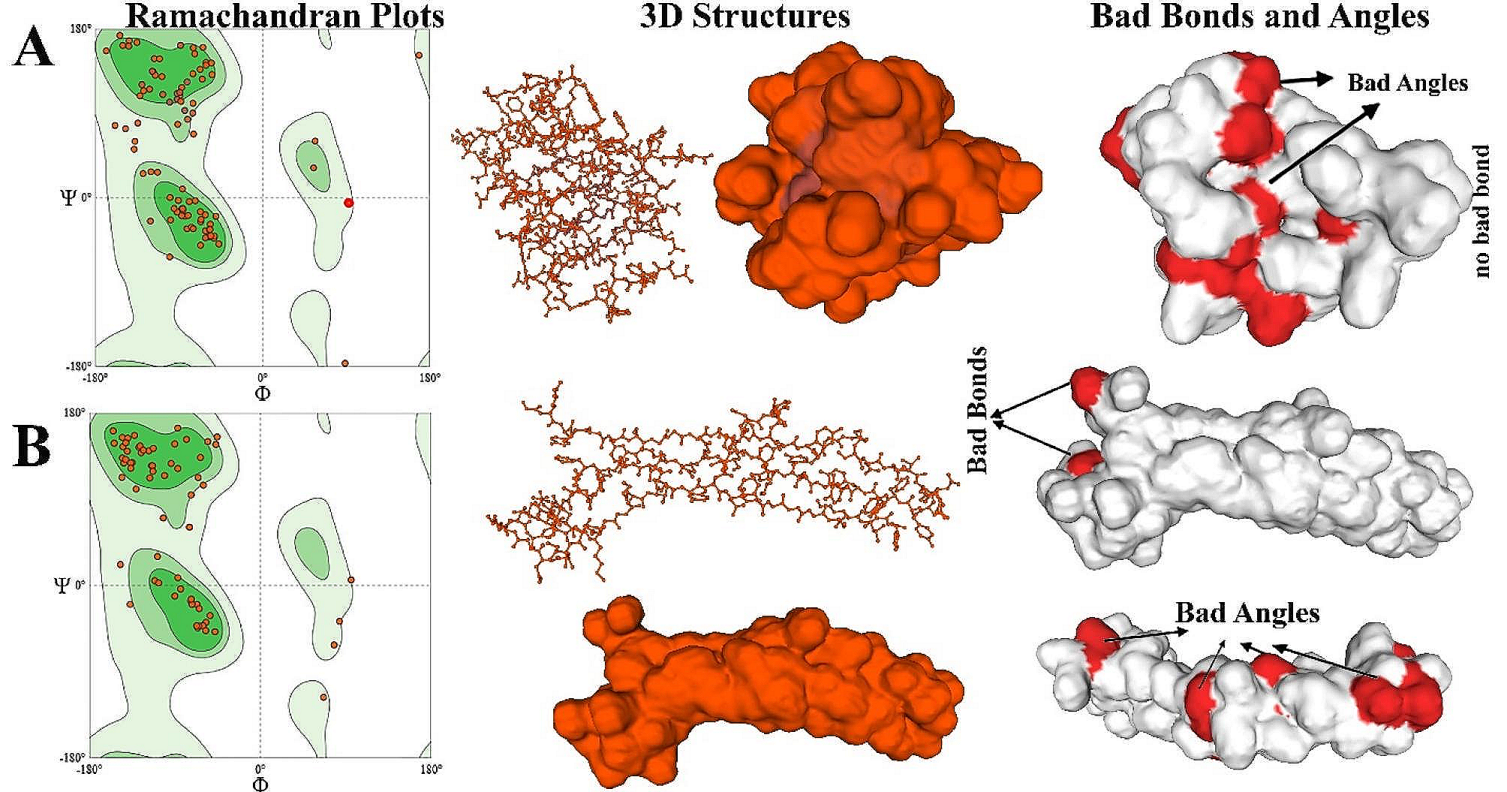
Ramachandran’s analysis. (**A**) vaccine-1 construct had no bad bond, 14 Bad Angles (**B**) vaccine-2 construct had 2 bad bonds in position of B357 ASP, B326 VAL and 14 Bad Angles

We made use of internet servers to assess the secondary structural aspects. Based on PSIPRED service projections, the final vaccine-1 construct would have 30.52% α-helix, 16.13% β-strand, and 53.13% random coils; the vaccine-2 construct would have 29.85% α-helix, 15.71% β-strand, and 51.47% random coils among its constituent parts. Ramachandran’s analysis shows that vaccine-1 has no bad bonds (out of 885). However, table S4 (https://s25.picofile.com/file/8454143276/S4_TABLE.docx.html) shows that there are 14 bad angles (A196 ALA, A149 ASP), (A207 ASP-A208 ASP), A155 PRO, (A151 THR-A152 MET), (A154 ALA-A155 PRO), (A265 TYR-A266 PRO, A208 ASP, A220 SER, A241 HIS, A240 PHE, A179 ASP). The results of Ramachandran’s study indicate that vaccine-2 contains two bad bonds (out of 696 bonds) and fourteen bad angles (B328 THR, B326 VAL, B302 TYR, B316 PHE, B356 GLN, B377 ASP, B387 PHE, B329 ASP, B319 TYR, B357 ASP, B313 ASP, B373 LYS, B338 LYS, (B338 LYS-B339 GLY), B328 THR, B326 VAL, and B357 ASP) (Fig. 5).

**Fig. 6:**
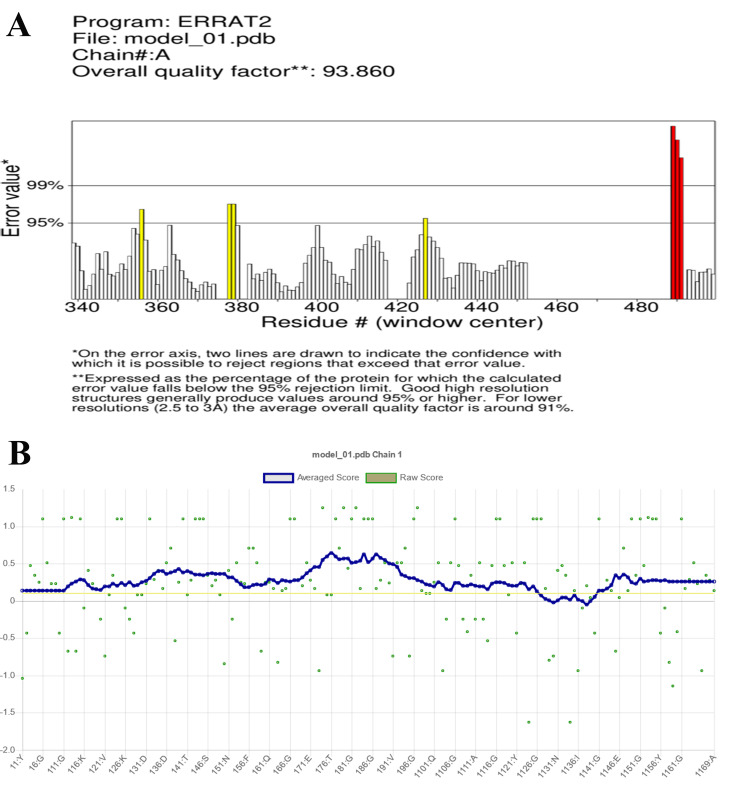
(**A**) The ERRAT results showed an overall quality factor of 93.8596. (**B**) at least 80% of the amino acids have scored > = 0.1 in the 3D/1D profile of Verify 3D

**Fig. 7:**
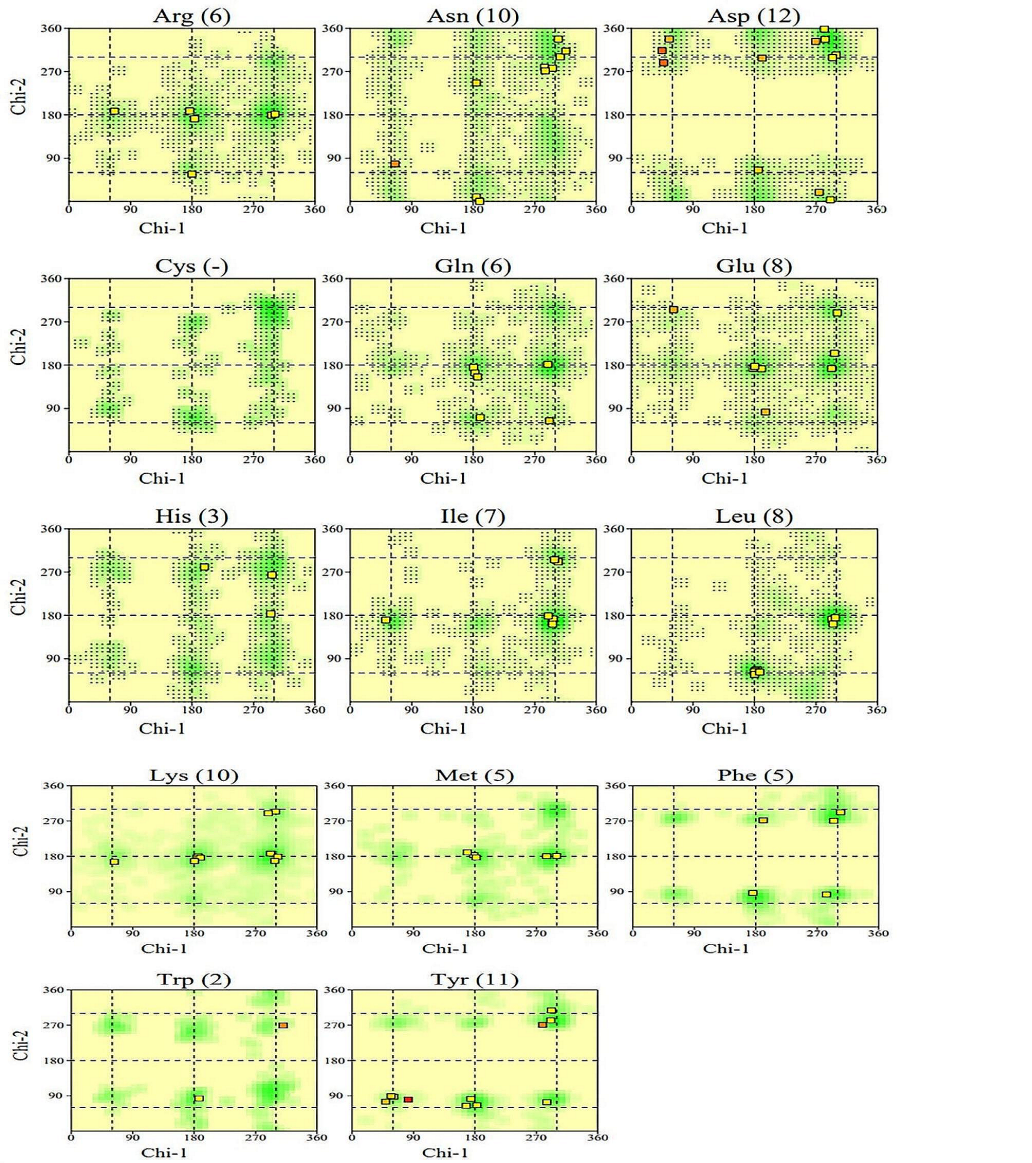
All-residue chi1-chi2 plots. Numbers of residues are shown in brackets. Those in unfavorable conformations (score < -3.00) are labelled. Shading shows favorable conformations as obtained from an analysis of 163 structures at resolution 2.0 A or better

The quality of Vaccination 1’s third structure was examined using validation techniques like ERRAT and Verify 3D. Figure 6A of the ERRAT findings demonstrated an average quality factor of 93.8596. Furthermore, in the 3D/1D profile of Verify 3D, at least 80% of the amino acids scored > = 0.1, and 91.72% of the residues had an average 3D-1D score > = 0.1, indicating that the third structure was approved (Fig. 6B). However, All-residue chi1-chi2 plots and All-residue Ramachandran plots both passed (Fig. 7). Figure 7 displayed the chi1-chi2 plots for each residual. Within parenthesis, the total amount of residues is shown. Labels were applied to those with poor combinations (scoring < -3.00). Positive conformations found via examination of 163 compounds at 2.0 A resolution or above are shown by shading (Fig. 7).

**Fig. 8:**
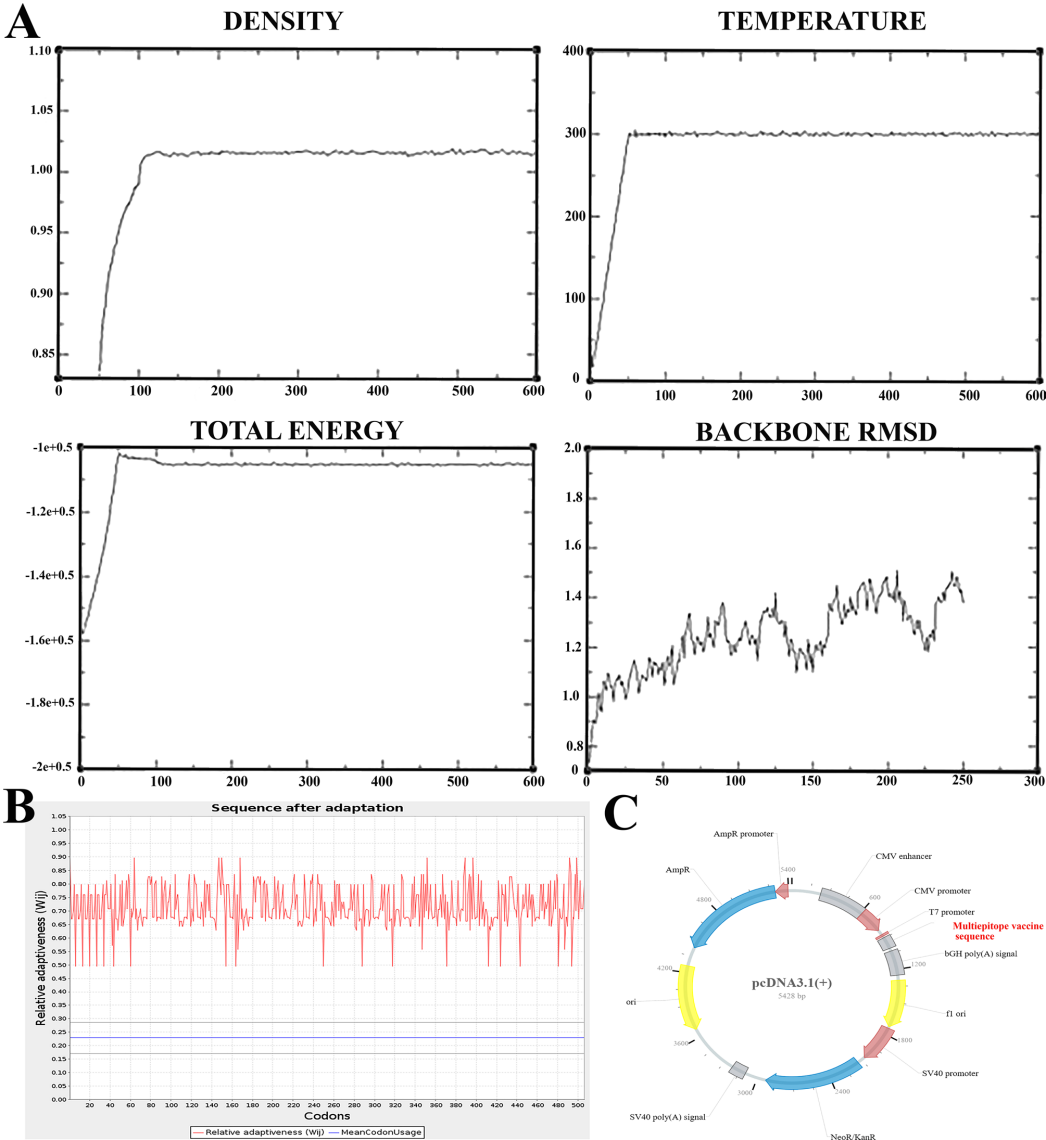
(**A**) Graphs generated from molecular dynamics (MD) simulation trajectories can be used to analyses a system’s structural stability. The density, temperature and total energy plots have all converged by the end of our equilibration period. The RMSD, while seeming to begin to level, does not have converged entirely but is acceptable. (**B**) JCAT results showed that the vaccine sequence was suitable for mouse muscle after adaptation with GC-Content of 6706%. (**C**) Map of pcDNA3.1(+) showing the multi-epitope vaccine locus

Molecular dynamics (MD) simulation trajectories can yield plots useful for assessing a system’s structural stability. By the end of our equilibrium period, the density, temperature, and total energy graphs had reached a state of convergence, suggesting the stability of the vaccine structure. As our equilibration time concludes, the density, temperature, and total energy graphs have all converged. Even though it seems to be leveling off, the RMSD is still tolerable and has not entirely converged (Fig. 8A). The JCAT findings demonstrated that, with adaptation, the vaccination sequence was appropriate for mouse muscle. Furthermore, as shown in Fig. 8B, the enhanced sequence’s GC content was similar to 67.06192358366272, and its CAI value was equal to 0.70980977263218006. Once it was chosen, the vaccine gene sequence of choice was introduced into the pcDNA3.1(+) vaccine construct. This project’s route map is displayed in Fig. 8C.

**Fig. 9:**
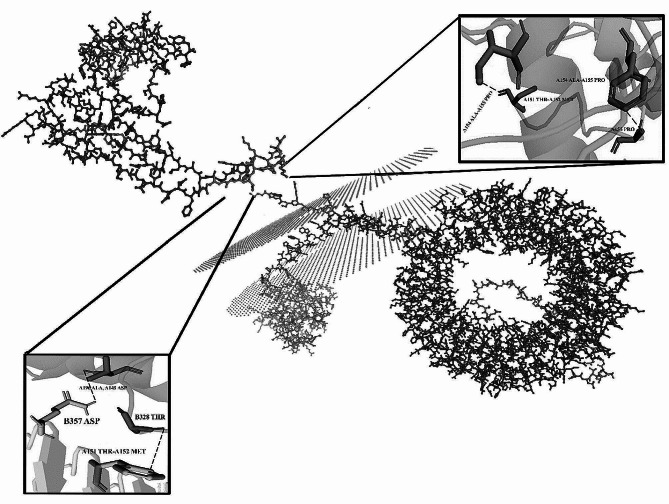
Analysis of molecular docking between the TLR-9 receptor and vaccine epitope. The results showed a strong binding between the TLR-9 receptor and the vaccine construct

Figure 9 depicts the molecular docking analysis of the TLR-9 receptor and vaccination epitope. The proposed vaccine was subjected to molecular docking to TLR-9 (PDB ID-3WPF) utilizing the ClusPro v 2.0 and Patchdock web servers. Consequently, ClusPro v 2.0 produced 29 structures for each docking. In both instances, the model with the highest binding affinity and lowest intermolecular energy was chosen. The docking process with TLR9 resulted in the lowest energy score of -1163, as shown in Fig. 9. The toxicity of the final epitopes in the vaccine structure was predicted and the results showed the non-toxicity of the vaccine structure (Table S5).

## Discussion

The use of whole organisms is a fundamental aspect of conventional vaccine manufacturing techniques, which have the potential to induce allergic responses and inadvertent antigen exposure [17]. Polypeptide vaccinations provide a potential solution to the issue of allergic reactions by eliminating the possibility of such reactions. These vaccines contain short segments of immunogenic peptides and induce robust and specific immune responses [18]. The prevalence of immunizations against infectious illnesses is on the rise and is very effective. In order to ensure the effectiveness of vaccines, the preclinical and clinical stages of vaccine development need the use of intricate, time-consuming, and costly in vivo and in vitro methodologies [19, 20].

The efficiency of in silico vaccine development is increasing due to advancements in bioinformatics and immunoinformatic [21]. This phenomenon results in a decline in In vitro testing procedures. The advancement of a multiepitope vaccine targeting K. pneumoniae was significantly facilitated by an in vitro investigation. The primary benefit of this technology is its ability to detect many candidate vaccinations without spreading bacterial illnesses across a wet laboratory [22]. Several instances of how vaccinosis has been used to construct a multi-epitope-based vaccination method include Acinetobacter baumannii [16], MERS-CoV, HIV, and SARS-CoV-2 [22–25].

Klebsiella is responsible for many diseases, including potentially fatal infections affecting the liver, circulation, and lungs. The absence of an FDA-approved vaccination that effectively prevents Klebsiella infections necessitates the urgent proposal of new vaccine candidates for immune protection studies [26]. The standard methodology for vaccine design is characterized by its high cost and significant time investment. Computational techniques can accelerate vaccine development by identifying potent vaccine candidates versus Klebsiella [27]. K. pneumoniae, being a diverse organism, continuously uncovers novel serotypes. Further research is required to identify a potential vaccine candidate to target a diverse array of K. pneumoniae effectively isolates [28].

The current work is unique in that it utilizes genomes to uncover a widely recognized vaccination candidate for many strains of K. pneumoniae rather than concentrating on a single organism. This kind of study is often called “pan-genome reverse vaccinology.” When developing a vaccine candidate, it is necessary to consider many microbial strains [29]. Researchers used this technique to clone and evaluate eight strains of group B streptococcus to identify vaccine candidates with high efficacy [30]. A methodology that integrates wet and dry laboratories was used to select possible vaccine candidates against diseases by analyzing the genome of a single strain. This approach facilitated the development of novel vaccine candidates targeting the bacteria [31].

This methodology has undergone testing against bacterial pathogens and a diverse range of other hazardous species. The efficiency of this strategy against the protozoan Leishmania was established by researchers using proteome screening. This was achieved using relevant properties such as subcellular localization, non-homology to the protein database, and ligand binding with MHC [32]. Another approach included the selection of a well-known antigenic spike peptide from the SARS coronavirus and subsequent analysis of its sequence to find the regions with the highest antigenicity. The decrease in energy improved these areas, resulting in increased antigenic characteristics [33]. Eleven thermodynamically stable epitopes, which exhibit similarity to natural proteins, were identified using molecular modeling investigations utilizing glycoconjugate addition on 14 epitopes derived from *Schistosoma mansoni*. The potential use of these epitopes as vaccine targets against schistosomiasis has been explored in wet laboratory trials [34].

A web-based approach was used to identify candidates from a pool of 10 surface-exposed adhesion molecules of Staphylococcus aureus known to cause endocarditis. This technique included the search for an epitope that exhibited conservation across different bacterial strains and showed the ability to induce humoral and cell-mediated protection [35]. With the continuous identification of whole genome sequences of disease-causing microorganisms and the genomes of specific surface antigens, it is now possible to rapidly test a specific antigen or group of proteins using a simple technical application. Reverse vaccination is a cost-effective strategy due to the extensive accessibility of genetic data and most screening tools. The reversal of vaccination has the potential to effectively cure a wide range of bacterial, fungal, viral, and parasitic diseases. The BLAST technology used in this approach applies to a wide range of organisms, including fungi and viruses. The analysis of input sequences in the IEDB B cell epitope identification software does not consider the kind of pathogen. Cello protein, a protein localization instrument, may be used to forecast the subcellular positioning of proteins [36].

In addition to its many benefits, reverse vaccination has significant drawbacks. The availability of complete genetic information about the pathogen is crucial for verifying vaccination candidates, while in vivo experiments should validate particular antigens [37, 38]. The study’s primary aim was to ascertain the proteins of K. pneumoniae that exhibit the highest degree of conservation and potential antigenicity via web-based bioinformatics methodologies. The pathogenicity and antigenic potential of pneumococcal surface antigens have been extensively investigated in animal experiments. Proteins such as pspA and pspC have been shown to promote resistance in mouse models [39].

Recombinant pneumococcal glycolytic proteins, including GAPDH (Glyceraldehyde 3-phosphate dehydrogenase) and FBA [40], are produced inside Escherichia coli and elicit the production of antibodies that exhibit specificity towards various serotypes. Research [41] shows an observed interaction between pneumococcal serotypes and vaccinations targeting choline-binding enzymes (CBPs). Following the administration of spuA and phpA vaccines [42], the body generates antibodies against pneumococci. The study utilized EAAAK, GGGS, and HEYGAEALERAG linkers to establish connections between various vaccine components. These linkers are crucial in determining both B and T cells’ 3D structure and subsequent exposure [43, 44]. The “EAAAK” linker attached the Adjuvant to vaccination sequences. This linker is an inflexible linker, which offers some advantages compared to flexible linkers. The “EAAAK” linker in a bi-functional fusion protein facilitates the efficient segregation of functional domains [45]. The HTL epitopes were connected using the “GGGS” linker. This linker can trigger HTL (helper T lymphocyte) responses, essential for creating multi-epitope vaccines. This linker inhibits the production of the majority of junctional epitopes [46]. The “HEYGAEALERAG” linker was utilized to join CTL epitopes. This linker serves as the target for both the lysosomal and proteasomal degradation processes, enhancing the presentation of epitopes.

## Conclusions

This study used a computational vaccine design approach to all *Klebsiella* genus species reference genomes. Three exceptionally conserved proteins and putative vaccination candidates that fit several vaccine candidacy traits were found. MrkD, RmpA, and iron-regulated outer membrane proteins are these proteins. Several highly rated epitopes from the epitope mapping phase of these proteins were used to create a multi-epitope peptide vaccination. The created vaccine ensemble also showed a prolonged binding configuration with the evaluated immune receptor and significant interaction energy. The time and expense of developing vaccines might be significantly reduced using computer-aided design. The process can also aid in identifying the most suitable vaccine candidates for examination. The study’s results are promising, but further research is needed to confirm the vaccine architecture’s biological efficacy. Despite its early stages, the present research is more economical and time-efficient than a typical method for screening potential vaccines; nonetheless, a biological test is still required to confirm the screening results.

### Electronic supplementary material

Below is the link to the electronic supplementary material.

Supplementary Material 1

## Data Availability

The data generated or analyzed during this study are included in this article and its additional materials.

## Abbreviations

AMR: antimicrobial resistance
CDC: Center for Disease Control and Prevention
ESBLs: extended-spectrum β-lactamases
SPC: capsular polysaccharide
RV: reverse vaccinology
PGRV: pan-genome-based RV

## References

[1] Dey J, Mahapatra SR, Raj TK, Misra N, Suar M. Identification of potential flavonoid compounds as antibacterial therapeutics against *Klebsiella pneumoniae* infection using structure-based virtual screening and molecular dynamics simulation. Mol Divers 2023 Oct 6:1–8. 10.1007/s11030-023-10738-z 37801217

[2] Asadipour E, Asgari M, Mousavi P, Piri-Gharaghie T, Ghajari G, Mirzaie A. Nano‐Biotechnology and challenges of Drug Delivery System in Cancer Treatment Pathway. Volume 1. Chemistry & Biodiversity; 2023 Mar. p. e202201072. 10.1002/cbdv.202201072 36857487

[3] Mancuso G, Midiri A, Gerace E, Biondo C (2021). Bacterial antibiotic resistance: the most critical pathogens. Pathogens.

[4] Liao JX, Appaneal HJ, Menon A, Lopes V, LaPlante KL, Caffrey AR. Decreasing Antibiotic Resistance trends nationally in Gram-Negative Bacteria across United States Veterans Affairs Medical Centers, 2011–2020. Infect Dis Therapy 2023 Jun 16:1–4. 10.1007/s40121-023-00827-9 PMC10390413 37326931

[5] Dey J, Mahapatra SR, Singh PK, Prabhuswamimath SC, Misra N, Suar M. Designing of multi-epitope peptide vaccine against Acinetobacter baumannii through combined immunoinformatics and protein interaction–based approaches. Immunol Res. 2023 Apr;6:1–24. 10.1007/s12026-023-09374-4 PMC10078064 37022613

[6] Cillóniz C, Dominedò C, Torres A. Multidrug resistant gram-negative bacteria in community-acquired pneumonia. Annual Update in Intensive Care and Emergency Medicine 2019. 2019:459 – 75. 10.1186/s13054-019-2371-3 PMC6408800 30850010

[7] Dey J, Mahapatra SR, Raj TK, Kaur T, Jain P, Tiwari A, Patro S, Misra N, Suar M (2022). Designing a novel multi-epitope vaccine to evoke a robust immune response against pathogenic multidrug-resistant Enterococcus faecium bacterium. Gut Pathogens.

[8] Khan AU, Maryam L, Zarrilli R (2017). Structure, genetics and worldwide spread of New Delhi metallo-β-lactamase (NDM): a threat to public health. BMC Microbiol.

[9] Chokshi A, Sifri Z, Cennimo D, Horng H (2019). Global contributors to antibiotic resistance. J Global Infect Dis.

[10] Dey J, Mahapatra SR, Lata S, Patro S, Misra N, Suar M (2022). Exploring *Klebsiella pneumoniae* capsule polysaccharide proteins to design multiepitope subunit vaccine to fight against pneumonia. Expert Rev Vaccines.

[11] Mahapatra SR, Dey J, Raj TK, Kumar V, Ghosh M, Verma KK, Kaur T, Kesawat MS, Misra N, Suar M (2022). The potential of plant-derived secondary metabolites as novel drug candidates against *Klebsiella pneumoniae*: molecular docking and simulation investigation. South Afr J Bot.

[12] Adu-Bobie J, Capecchi B, Serruto D, Rappuoli R, Pizza M (2003). Two years into reverse vaccinology. Vaccine.

[13] Fernandes LG, Teixeira AF, Antonio Filho FS, Souza GO, Vasconcellos SA, Heinemann MB, Romero EC, Nascimento AL (2017). Immune response and protective profile elicited by a multi-epitope chimeric protein derived from Leptospira interrogans. Int J Infect Dis.

[14] Narang PK, Dey J, Mahapatra SR, Roy R, Kushwaha GS, Misra N, Suar M, Raina V (2022). Genome-based identification and comparative analysis of enzymes for carotenoid biosynthesis in microalgae. World J Microbiol Biotechnol.

[15] Ismail S, Shahid F, Khan A, Bhatti S, Ahmad S, Naz A, Almatroudi A, ul Qamar MT (2021). Pan-vaccinomics approach towards a universal vaccine candidate against WHO priority pathogens to address growing global antibiotic resistance. Comput Biol Med.

[16] Simbulan AM, Banico EC, Sira EM, Odchimar NM, Orosco FL (2024). Immunoinformatics-guided approach for designing a pan-proteome multi-epitope subunit vaccine against African swine fever virus. Sci Rep.

[17] Narang PK, Dey J, Mahapatra SR, Ghosh M, Misra N, Suar M, Kumar V, Raina V (2021). Functional annotation and sequence-structure characterization of a hypothetical protein putatively involved in carotenoid biosynthesis in microalgae. South Afr J Bot.

[18] Nel AE, Miller JF (2021). Nano-enabled COVID-19 vaccines: meeting the challenges of durable antibody plus cellular immunity and immune escape. ACS Nano.

[19] Ghattas M, Dwivedi G, Lavertu M, Alameh MG (2021). Vaccine technologies and platforms for infectious diseases: current progress, challenges, and opportunities. Vaccines.

[20] Choy RK, Bourgeois AL, Ockenhouse CF, Walker RI, Sheets RL, Flores J (2022). Controlled human infection models to accelerate Vaccine Development. Clin Microbiol Rev.

[21] Sudeshna Panda S, Dey J, Mahapatra SR, Kushwaha GS, Misra N, Suar M, Ghosh M (2022). Investigation on structural prediction of pectate lyase enzymes from different microbes and comparative docking studies with pectin: the economical waste from food industry. Geomicrobiol J.

[22] Kazi A, Chuah C, Majeed AB, Leow CH, Lim BH, Leow CY (2018). Current progress of immunoinformatics approach harnessed for cellular-and antibody-dependent vaccine design. Pathogens Global Health.

[23] Mahapatra SR, Dey J, Jaiswal A, Roy R, Misra N, Suar M (2022). Immunoinformatics-guided designing of epitope-based subunit vaccine from Pilus assembly protein of Acinetobacter baumannii bacteria. J Immunol Methods.

[24] Sahoo P, Dey J, Mahapatra SR, Ghosh A, Jaiswal A, Padhi S, Prabhuswamimath SC, Misra N, Suar M (2022). Nanotechnology and COVID-19 convergence: toward new planetary health interventions against the pandemic. OMICS.

[25] Ribeiro SP, Mattaraia VG, Almeida RR, Valentine EJ, Sales NS, Ferreira LC, Sa-Rocha LC, Jacintho LC, Santana VC, Sidney J, Sette A (2022). A promiscuous T cell epitope-based HIV vaccine providing redundant population coverage of the HLA class II elicits broad, polyfunctional T cell responses in nonhuman primates. Vaccine.

[26] Mahapatra SR, Dey J, Raj TK, Misra N, Suar M (2023). Designing a next-generation multiepitope-based vaccine against *Staphylococcus aureus* using reverse vaccinology approaches. Pathogens.

[27] Umar A, Haque A, Alghamdi YS, Mashraqi MM, Rehman A, Shahid F, Khurshid M, Ashfaq UA (2021). Development of a candidate multi-epitope subunit vaccine against *Klebsiella* aerogenes: subtractive proteomics and immuno-informatics approach. Vaccines.

[28] Ahmad TA, El-Sayed LH, Haroun M, Hussein AA, El Sayed H (2012). Development of immunization trials against Klebsiella pneumoniae. Vaccine.

[29] Jalal K, Khan K, Ahmad D, Hayat A, Basharat Z, Abbas MN, Alghamdi S, Almehmadi M, Sahibzada MU (2021). Pan-genome reverse vaccinology approach for the design of multi-epitope vaccine construct against Escherichia albertii. Int J Mol Sci.

[30] Maione D, Margarit I, Rinaudo CD, Masignani V, Mora M, Scarselli M, Tettelin H, Brettoni C, Iacobini ET, Rosini R, D’Agostino N (2005). Identification of a universal Group B *streptococcus* vaccine by multiple genome screen. Science.

[31] Wizemann TM, Heinrichs JH, Adamou JE, Erwin AL, Kunsch C, Choi GH, Barash SC, Rosen CA, Masure HR, Tuomanen E, Gayle A (2001). Use of a whole genome approach to identify vaccine molecules affording protection against *Streptococcus* pneumoniae infection. Infect Immun.

[32] John L, John GJ, Kholia T (2012). A reverse vaccinology approach for the identification of potential vaccine candidates from *Leishmania* spp. Appl Biochem Biotechnol.

[33] Chukwudozie OS, Gray CM, Fagbayi TA, Chukwuanukwu RC, Oyebanji VO, Bankole TT, Adewole RA, Eze DM. Immuno-Informatics design of a multimeric epitope peptide based Vaccine Targeting SARS-CoV-2 Spike Glycoprotein (preprint). 2020. 10.1371/journal.pone.0248061 PMC7968690 33730022

[34] de Oliveira Lopes D, de Oliveira FM, do, Vale Coelho IE, de Oliveira Santana KT, Mendonça FC, Taranto AG, dos Santos LL, Miyoshi A, de Carvalho Azevedo VA, Comar M Jr. Identification of a vaccine against schistosomiasis using bioinformatics and molecular modeling tools. Infection, Genetics and Evolution. 2013;20:83–95. 10.1016/j.meegid.2013.08.007 23973434

[35] Brady RA, Leid JG, Camper AK, Costerton JW, Shirtliff ME (2006). Identification of *Staphylococcus aureus* proteins recognized by the antibody-mediated immune response to a biofilm infection. Infect Immun.

[36] Shen HB, Chou KC. Virus-PLoc: A fusion classifier for predicting the subcellular localization of viral proteins within host and virus‐infected cells. Biopolymers: Original Research on Biomolecules. 2007;85(3):233 – 40. 10.1002/bip.20640 17120237

[37] Tregoning JS, Flight KE, Higham SL, Wang Z, Pierce BF (2021). Progress of the COVID-19 vaccine effort: viruses, vaccines and variants versus efficacy, effectiveness and escape. Nat Rev Immunol.

[38] Ghajari G, Nabiuni M, Amini E (2021). The association between testicular toxicity induced by Li2Co3 and protective effect of Ganoderma lucidum: alteration of Bax & c-Kit genes expression. Tissue Cell.

[39] Ghajari G, Heydari A, Ghorbani M (2023). Mesenchymal stem cell-based therapy and female infertility: limitations and advances. Curr Stem Cell Res Therapy.

[40] Gani Z, Boradia VM, Kumar A, Patidar A, Talukdar S, Choudhary E, Singh R, Agarwal N, Raje M, Iyengar Raje C (2021). Mycobacterium tuberculosis glyceraldehyde-3‐phosphate dehydrogenase plays a dual role—As an adhesin and as a receptor for plasmin (ogen). Cell Microbiol.

[41] Gosink KK, Mann ER, Guglielmo C, Tuomanen EI, Masure HR (2000). Role of novel choline binding proteins in virulence of *Streptococcus* pneumoniae. Infect Immun.

[42] Zhang Y, Masi AW, Barniak V, Mountzouros K, Hostetter MK, Green BA (2001). Recombinant PhpA protein, a unique histidine motif-containing protein from *Streptococcus* pneumoniae, protects mice against intranasal pneumococcal challenge. Infect Immun.

[43] Mahmoudvand S, Esmaeili Gouvarchin Ghaleh H, Jalilian FA, Farzanehpour M, Dorostkar R (2023). Design of a multi-epitope‐based vaccine consisted of immunodominant epitopes of structural proteins of SARS‐CoV‐2 using immunoinformatics approach. Biotechnol Appl Chem.

[44] Sanches RC, Tiwari S, Ferreira LC, Oliveira FM, Lopes MD, Passos MJ, Maia EH, Taranto AG, Kato R, Azevedo VA, Lopes DO (2021). Immunoinformatics design of multi-epitope peptide-based vaccine against Schistosoma mansoni using transmembrane proteins as a target. Front Immunol.

[45] Ayyagari VS, TC V, Srirama KAP (2022). Design of a multi-epitope-based vaccine targeting M-protein of SARS-CoV2: an immunoinformatics approach. J Biomol Struct Dynamics.

[46] Livingston B, Crimi C, Newman M, Higashimoto Y, Appella E, Sidney J, Sette A (2002). A rational strategy to design multiepitope immunogens based on multiple th lymphocyte epitopes. J Immunol.

