# Supplementary material for: A novel chimeric vaccine containing multiple epitopes for simulating robust immune activation against Klebsiella pneumoniae
Source: BMC Immunol. 2024 May 5;25:27. doi: 10.1186/s12865-024-00617-z (PMC11070107; doi:10.1186/s12865-024-00617-z)
Supplement: Supplementary file 1 — Supplementary Material 1 [file 12865_2024_617_MOESM1_ESM.docx]

**Supplementary materials**

**A Novel Chimeric Vaccine Containing Multiple Epitopes for Simulating Robust Immune Activation Against *Klebsiella pneumoniae***

Morteza Hakimian^1^, Abbas Doosti ^2,*^, Ali Sharifzadeh^2,3^

^1^Department of Biology, Faculty of Basic Sciences, Shahrekord Branch, Islamic Azad University, Shahrekord, Iran

^2^Biotechnology Research Center, Shahrekord Branch, Islamic Azad University, Shahrekord, Iran.

^3^Department of Microbiology, Faculty of Veterinary Medicine, Islamic Azad University, Shahrekord Branch, Shahrekord,Iran.

**Table S1.** Genome information for reference (*Klebsiella pneumoniae* subsp. pneumoniae HS11286) and representative genomes. Genome sequences used in this study are listed in the table.

| Type | Name | RefSeq | INSDC | Size (Mb) | GC% | Protein | rRNA | tRNA | Other RNA | Gene |
| --- | --- | --- | --- | --- | --- | --- | --- | --- | --- | --- |
| Chr | - | [NC_016845.1](https://www.ncbi.nlm.nih.gov/nuccore/NC_016845.1) | [CP003200.1](https://www.ncbi.nlm.nih.gov/nuccore/CP003200.1) | 5.33 | 57.5 | [5,316](https://www.ncbi.nlm.nih.gov/genome/browse/#!/proteins/815/168877\|Klebsiella pneumoniae subsp. pneumoniae HS11286/chromosome/) | 25 | 62 | 1 | 5,404 |
| Plsm | pKPHS1 | [NC_016838.1](https://www.ncbi.nlm.nih.gov/nuccore/NC_016838.1) | [CP003223.1](https://www.ncbi.nlm.nih.gov/nuccore/CP003223.1) | 0.12 | 49.5 | [141](https://www.ncbi.nlm.nih.gov/genome/browse/#!/proteins/815/168877\|Klebsiella pneumoniae subsp. pneumoniae HS11286/plasmid pKPHS1/) | - | - | - | 141 |
| Plsm | pKPHS2 | [NC_016846.1](https://www.ncbi.nlm.nih.gov/nuccore/NC_016846.1) | [CP003224.1](https://www.ncbi.nlm.nih.gov/nuccore/CP003224.1) | 0.11 | 53.3 | [160](https://www.ncbi.nlm.nih.gov/genome/browse/#!/proteins/815/168877\|Klebsiella pneumoniae subsp. pneumoniae HS11286/plasmid pKPHS2/) | - | - | - | 160 |
| Plsm | pKPHS3 | [NC_016839.1](https://www.ncbi.nlm.nih.gov/nuccore/NC_016839.1) | [CP003225.1](https://www.ncbi.nlm.nih.gov/nuccore/CP003225.1) | 0.11 | 52.5 | [152](https://www.ncbi.nlm.nih.gov/genome/browse/#!/proteins/815/168877\|Klebsiella pneumoniae subsp. pneumoniae HS11286/plasmid pKPHS3/) | - | - | - | 152 |
| Plsm | pKPHS4 | [NC_016840.1](https://www.ncbi.nlm.nih.gov/nuccore/NC_016840.1) | [CP003226.1](https://www.ncbi.nlm.nih.gov/nuccore/CP003226.1) | 0 | 52.2 | [4](https://www.ncbi.nlm.nih.gov/genome/browse/#!/proteins/815/168877\|Klebsiella pneumoniae subsp. pneumoniae HS11286/plasmid pKPHS4/) | - | - | - | 4 |
| Plsm | pKPHS5 | [NC_016847.1](https://www.ncbi.nlm.nih.gov/nuccore/NC_016847.1) | [CP003227.1](https://www.ncbi.nlm.nih.gov/nuccore/CP003227.1) | 0 | 42.8 | [5](https://www.ncbi.nlm.nih.gov/genome/browse/#!/proteins/815/168877\|Klebsiella pneumoniae subsp. pneumoniae HS11286/plasmid pKPHS5/) | - | - | - | 5 |
| Plsm | pKPHS6 | [NC_016841.1](https://www.ncbi.nlm.nih.gov/nuccore/NC_016841.1) | [CP003228.1](https://www.ncbi.nlm.nih.gov/nuccore/CP003228.1) | 0 | 47.9 | [1](https://www.ncbi.nlm.nih.gov/genome/browse/#!/proteins/815/168877\|Klebsiella pneumoniae subsp. pneumoniae HS11286/plasmid pKPHS6/) | - | - | - | 1 |

**Table S2.** List of HLA allele used in this study to specify MHC allele sequence. The list of HLA alleles used in this study to ascertain the MHC variant sequence

| Allele | Length |  |
| --- | --- | --- |
| HLA-A*01:01 | 9 | 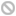 |
| HLA-A*01:01 | 10 | 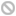 |
| HLA-A*02:01 | 9 | 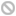 |
| HLA-A*02:01 | 10 | 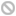 |
| HLA-A*02:03 | 9 | 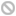 |
| HLA-A*02:03 | 10 | 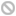 |
| HLA-A*02:06 | 9 | 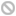 |
| HLA-A*02:06 | 10 | 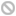 |
| HLA-A*03:01 | 9 | 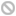 |
| HLA-A*03:01 | 10 | 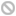 |
| HLA-A*11:01 | 9 | 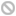 |
| HLA-A*11:01 | 10 | 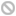 |
| HLA-A*23:01 | 9 | 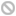 |
| HLA-A*23:01 | 10 | 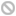 |
| HLA-A*24:02 | 9 | 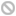 |
| HLA-A*24:02 | 10 | 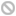 |
| HLA-A*26:01 | 9 | 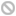 |
| HLA-A*26:01 | 10 | 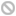 |
| HLA-A*30:01 | 9 | 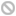 |
| HLA-A*30:01 | 10 | 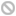 |
| HLA-A*30:02 | 9 | 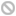 |
| HLA-A*30:02 | 10 | 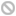 |
| HLA-A*31:01 | 9 | 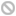 |
| HLA-A*31:01 | 10 | 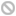 |
| HLA-A*32:01 | 9 | 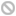 |
| HLA-A*32:01 | 10 | 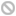 |
| HLA-A*33:01 | 9 | 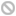 |
| HLA-A*33:01 | 10 | 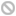 |
| HLA-A*68:01 | 9 | 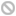 |
| HLA-A*68:01 | 10 | 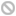 |
| HLA-A*68:02 | 9 | 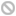 |
| HLA-A*68:02 | 10 | 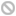 |
| HLA-B*07:02 | 9 | 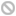 |
| HLA-B*07:02 | 10 | 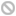 |
| HLA-B*08:01 | 9 | 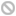 |
| HLA-B*08:01 | 10 | 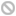 |
| HLA-B*15:01 | 9 | 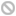 |
| HLA-B*15:01 | 10 | 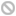 |
| HLA-B*35:01 | 9 | 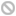 |
| HLA-B*35:01 | 10 | 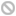 |
| HLA-B*40:01 | 9 | 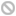 |
| HLA-B*40:01 | 10 | 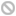 |
| HLA-B*44:02 | 9 | 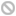 |
| HLA-B*44:02 | 10 | 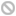 |
| HLA-B*44:03 | 9 | 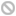 |
| HLA-B*44:03 | 10 | 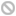 |
| HLA-B*51:01 | 9 | 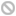 |
| HLA-B*51:01 | 10 | 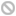 |
| HLA-B*53:01 | 9 | 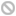 |
| HLA-B*53:01 | 10 | 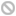 |
| HLA-B*57:01 | 9 | 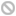 |
| HLA-B*57:01 | 10 | 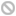 |
| HLA-B*58:01 | 9 | 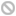 |
| HLA-B*58:01 | 10 |  |

**Table S3.** The population coverage investigation of the proteins MHC-I and class combined epitopes was submitted in bellow site. the results of the population coverage study for the RmpA protein MHCI and class combination epitopes, which showed 44.37% and 50.79% worldwide coverage, respectively

<https://s24.picofile.com/file/8454030850/S3_TABLE.docx.html>

## Table S3: Population Coverage Calculation Result for MrkD, Iron-regulated outer membrane proteins and RampA

## Population Coverage Calculation Result for MrkD

| population/area | Class I | | | Class II | | | Class combined | | |
| --- | --- | --- | --- | --- | --- | --- | --- | --- | --- |
|  | **coverage^a^** | **average_hit^b^** | **pc90^c^** | **coverage^a^** | **average_hit^b^** | **pc90^c^** | **coverage^a^** | **average_hit^b^** | **pc90^c^** |
| [World](http://tools.iedb.org/population/result/#World) | 54.01% | 0.86 | 0.22 | 0.0% | 0.0 | 0.0 | 54.01% | 0.86 | 0.22 |
|  |  |  |  |  |  |  |  |  |  |
| Average | **54.01** | **0.86** | **0.22** | **0.0** | **0.0** | **0.0** | **54.01** | **0.86** | **0.22** |
| Standard deviation | **0.0** | **0.0** | **0.0** | **0.0** | **0.0** | **0.0** | **0.0** | **0.0** | **0.0** |

^a^ projected population coverage
^b^ average number of epitope hits / HLA combinations recognized by the population
^c^ minimum number of epitope hits / HLA combinations recognized by 90% of the population

| Epitope | Coverage | HLA allele (genotypic frequency (%)) | | | | | | | Total HLA hits |
| --- | --- | --- | --- | --- | --- | --- | --- | --- | --- |
|  | **Class I** | **HLA-A*01:01 (10.09)** | **HLA-A*24:02 (12.59)** | **HLA-A*30:02 (1.36)** | **HLA B*15:01 (5.65)** | **HLA-B*40:01 (5.22)** | **HLA-B*44:03 (4.17)** | **HLA-B*57:01 (2.58)** |  |
| Epitope #1: CTRLSSPTV | 21.38% | - | + | - | - | - | - | - | **1** |
| Epitope #2: TMSAPGGASY | 8.44% | - | - | - | + | - | - | - | **1** |
| Epitope #3: WESGGNPIL | 7.81% | - | - | - | - | + | - | - | **1** |
| Epitope #4: GSVILTRDW | 3.90% | - | - | - | - | - | - | + | **1** |
| Epitope #5: MSAPGGASY | 17.34% | + | - | - | - | - | - | - | **1** |
| Epitope #6: TMSAPGGASY | 8.44% | - | - | - | + | - | - | - | **1** |
| Epitope #7: MSLRKLLTLF | 3.90% | - | - | - | - | - | - | + | **1** |
| Epitope #8: GEVESHMIF | 6.27% | - | - | - | - | - | + | - | **1** |
| Epitope #9: RYITIPLHAR | 6.27% | - | - | - | - | - | + | - | **1** |
| Epitope #10: RLNNQETRY | 2.43% | - | - | + | - | - | - | - | **1** |
| Epitope set | **54.01%** | **1** | **1** | **1** | **2** | **1** | **2** | **2** | **10** |

+ : restricted
- : not restricted
shaded column : genotypic frequency of this allele is 0 (zero)

## Population Coverage Calculation Result iron

| population/area | Class I | | | Class II | | | Class combined | | |
| --- | --- | --- | --- | --- | --- | --- | --- | --- | --- |
|  | **coverage^a^** | **average_hit^b^** | **pc90^c^** | **coverage^a^** | **average_hit^b^** | **pc90^c^** | **coverage^a^** | **average_hit^b^** | **pc90^c^** |
| [World](http://tools.iedb.org/population/result/#World) | 48.66% | 0.75 | 0.19 | 0.0% | 0.0 | 0.0 | 48.66% | 0.75 | 0.19 |
|  |  |  |  |  |  |  |  |  |  |
| Average | **48.66** | **0.75** | **0.19** | **0.0** | **0.0** | **0.0** | **48.66** | **0.75** | **0.19** |
| Standard deviation | **0.0** | **0.0** | **0.0** | **0.0** | **0.0** | **0.0** | **0.0** | **0.0** | **0.0** |

^a^ projected population coverage
^b^ average number of epitope hits / HLA combinations recognized by the population
^c^ minimum number of epitope hits / HLA combinations recognized by 90% of the population

population: World

| **MHC class** | **Coverage** | **Average hit** | **PC90** |
| --- | --- | --- | --- |
| I | 48.66% | 0.75 | 0.19 |

## World

| Epitope | Coverage | HLA allele (genotypic frequency (%)) | | | | | | | Total HLA hits |
| --- | --- | --- | --- | --- | --- | --- | --- | --- | --- |
|  | **Class I** | **HLA-A*11:01 (8.99)** | **HLA-A*23:01 (3.06)** | **HLA-A*26:01 (3.28)** | **HLA-B*07:02 (8.65)** | **HLA-B*15:01 (5.65)** | **HLA-B*40:01 (5.22)** | **HLA-B*57:01 (2.58)** |  |
| Epitope #1: EQQVQGGKEF | 5.82% | - | - | + | - | - | - | - | **1** |
| Epitope #2: QSDEDSIIV | 8.44% | - | - | - | - | + | - | - | **1** |
| Epitope #3: VSGGVRYQW | 3.90% | - | - | - | - | - | - | + | **1** |
| Epitope #4: NTVDFIGSY | 5.82% | - | - | + | - | - | - | - | **1** |
| Epitope #5: VYYRDESLTF | 5.43% | - | + | - | - | - | - | - | **1** |
| Epitope #6: IEIDDNRQL | 7.81% | - | - | - | - | - | + | - | **1** |
| Epitope #7: GSYALPVGK | 15.53% | + | - | - | - | - | - | - | **1** |
| Epitope #8: QTDGRWQKW | 3.90% | - | - | - | - | - | - | + | **1** |
| Epitope #9: HPLLLASTL | 12.78% | - | - | - | + | - | - | - | **1** |
| Epitope #10: ETFNANQMF | 5.82% | - | - | + | - | - | - | - | **1** |
| Epitope set | **48.66%** | **1** | **1** | **3** | **1** | **1** | **1** | **2** | **10** |

+ : restricted
- : not restricted
shaded column : genotypic frequency of this allele is 0 (zero)

## Population Coverage Calculation Result rampA

| population/area | Class I | | | Class II | | | Class combined | | |
| --- | --- | --- | --- | --- | --- | --- | --- | --- | --- |
|  | **coverage^a^** | **average_hit^b^** | **pc90^c^** | **coverage^a^** | **average_hit^b^** | **pc90^c^** | **coverage^a^** | **average_hit^b^** | **pc90^c^** |
| [World](http://tools.iedb.org/population/result/#World) | 44.37% | 0.72 | 0.18 | 0.0% | 0.0 | 0.0 | 44.37% | 0.72 | 0.18 |
|  |  |  |  |  |  |  |  |  |  |
| Average | **44.37** | **0.72** | **0.18** | **0.0** | **0.0** | **0.0** | **44.37** | **0.72** | **0.18** |
| Standard deviation | **0.0** | **0.0** | **0.0** | **0.0** | **0.0** | **0.0** | **0.0** | **0.0** | **0.0** |

^a^ projected population coverage
^b^ average number of epitope hits / HLA combinations recognized by the population
^c^ minimum number of epitope hits / HLA combinations recognized by 90% of the population

Population: World

| MHC class | Coverage | Average hit | PC90 |
| --- | --- | --- | --- |
| I | 44.37% | 0.72 | 0.18 |

## World

| Epitope | Coverage | HLA allele (genotypic frequency (%)) | | | | | | Total HLA hits |
| --- | --- | --- | --- | --- | --- | --- | --- | --- |
|  | **Class I** | **HLA-A*03:01 (9.77)** | **HLA-A*11:01 (8.99)** | **HLA-A*26:01 (3.28)** | **HLA-A*31:01 (3.02)** | **HLA-B*57:01 (2.58)** | **HLA-B*58:01 (2.26)** |  |
| Epitope #1: LTDDYFFYY | 15.53% | - | + | - | - | - | - | **1** |
| Epitope #2: KLYFAFLKK | 16.81% | + | - | - | - | - | - | **1** |
| Epitope #3: ITYEGVVNK | 15.53% | - | + | - | - | - | - | **1** |
| Epitope #4: GSLNMISQW | 3.90% | - | - | - | - | + | - | **1** |
| Epitope #5: KSIAIKHKR | 5.36% | - | - | - | + | - | - | **1** |
| Epitope #6: DVSGGGRFY | 5.82% | - | - | + | - | - | - | **1** |
| Epitope #7: KTLSCYQSK | 5.82% | - | - | + | - | - | - | **1** |
| Epitope #8: LNMISQWMW | 3.42% | - | - | - | - | - | + | **1** |
| Epitope set | **44.37%** | **1** | **2** | **2** | **1** | **1** | **1** | **8** |

+ : restricted
- : not restricted
shaded column : genotypic frequency of this allele is 0 (zero)

| Cluster Number | Peptide Number | Alignment | Position | Description | Peptide |
| --- | --- | --- | --- | --- | --- |
| 1 | Consensus | GGYKMLRGSLNMISQWMWSGKWGGYKMLRGSLNMIS | - | - | - |
| 1 | 1 | GGYKMLRGSLNMISQ--------------------- | 1 | seq15 | GGYKMLRGSLNMISQ |
| 1 | 2 | -------GSLNMISQW-------------------- | 8 | seq8 | GSLNMISQW |
| 1 | 3 | ---------LNMISQWMW------------------ | 10 | seq20 | LNMISQWMW |
| 1 | 4 | ------------------SGKWGGYKMLRGSLNMIS | 19 | seq17 | SGKWGGYKMLRGSLNMIS |
| 2 | Consensus | FHITYEGVVNKSIAIK | - | - | - |
| 2 | 1 | FHITYEGVVNKSIAI- | 1 | seq5 | FHITYEGVVNKSIAI |
| 2 | 2 | --ITYEGVVNK----- | 3 | seq6 | ITYEGVVNK |
| 2 | 3 | -------VVNKSIAIK | 8 | seq7 | VVNKSIAIK |
| 3 | Consensus | EKLYFAFLKKNVSRIVN | - | - | - |
| 3 | 1 | EKLYFAFLKKNVSRI-- | 1 | seq4, seq9 | EKLYFAFLKKNVSRI |
| 3 | 2 | -KLYFAFLKK------- | 2 | seq3 | KLYFAFLKK |
| 3 | 3 | --LYFAFLKKNVSRIVN | 3 | seq12 | LYFAFLKKNVSRIVN |
| 4 | Consensus | NIVLTDDYFFYYGLK | - | - | - |
| 4 | 1 | NIVLTDDYFFYYGLK | 1 | seq2 | NIVLTDDYFFYYGLK |
| 4 | 2 | ---LTDDYFFYY--- | 4 | seq1 | LTDDYFFYY |
| 5 | Consensus | DVSGGGRFYPKGCDYDIYVNMQGNVKNN | - | - | - |
| 5 | 1 | DVSGGGRFY------------------- | 1 | seq14 | DVSGGGRFY |
| 5 | 2 | --SGGGRFYPKGCDYDIYVNMQGNVKNN | 3 | seq16 | SGGGRFYPKGCDYDIYVNMQGNVKNN |
| 6 | Singleton | KSIAIKHKR | - | seq11 | KSIAIKHKR |
| 7 | Singleton | GGINEIKSQLKIEEKT | - | seq10 | GGINEIKSQLKIEEKT |
| 8 | Singleton | LQCLLKNGGINEIKS | - | seq21 | LQCLLKNGGINEIKS |
| 9 | Singleton | KEMVDERWLM | - | seq13 | KEMVDERWLM |
| 10 | Singleton | FFYYGLKQLTGLPLF | - | seq18 | FFYYGLKQLTGLPLF |
| 11 | Singleton | KTLSCYQSK | - | seq19 | KTLSCYQSK |
| 12 | Singleton | SRIVNHYPRLTKK | - | seq22 | SRIVNHYPRLTKK |

**Table S4.** Ramachandran's analysis of vaccines was submitted in bellow site. there are 14 bad angles (A196 ALA, A149 ASP), (A207 ASP-A208 ASP), A155 PRO, (A151 THR-A152 MET), (A154 ALA-A155 PRO), (A265 TYR-A266 PRO, A208 ASP, A220 SER, A241 HIS, A240 PHE, A179 ASP). The results of Ramachandran's study indicate that vaccine-2 contains two bad bonds (out of 696 bonds) and fourteen bad angles (B328 THR, B326 VAL, B302 TYR, B316 PHE, B356 GLN, B377 ASP, B387 PHE, B329 ASP, B319 TYR, B357 ASP, B313 ASP, B373 LYS, B338 LYS, (B338 LYS-B339 GLY), B328 THR, B326 VAL, and B357 ASP)

<https://s25.picofile.com/file/8454143276/S4_TABLE.docx.html>.

## Table S4: Ramachandran's analysis

## Vaccine-1

| MolProbity Score | 2.31 | --- |
| --- | --- | --- |
| Clash Score | 13.82 | (A184 VAL-A190 PHE) |
| Ramachandran Favoured | 88.29% | --- |
| Ramachandran Outliers | 2.70% | A155 LYS, A210 LYS, A167 SER |
| Rotamer Outliers | 1.23% | A185 LEU |
| C-Beta Deviations | 6 | A185 LEU, A183 ILE, A190 PHE, A167 SER, A229 ASN, A152 GLN |
| Bad Bonds | 0 / 832 | --- |
| Bad Angles | 36 / 1118 | A211 ASN, A183 ILE, A185 LEU, A191 PHE, A190 PHE, (A183 ILE-A184 VAL), A229 ASN, (A134 ALA-A135 PRO), (A169 GLU-A170 ASP), A189 TYR, A184 VAL, (A189 TYR-A190 PHE), (A197 GLY-A198 GLY), (A194 GLY-A195 LEU), A223 THR, A167 SER, (A184 VAL-A185 LEU), (A165 ALA-A166 GLN), A238 SER, (A182 ASN-A183 ILE), (A242 ASN-A243 ILE), A221 HIS |

## Vaccine-2

| MolProbity Score | 2.07 |  |
| --- | --- | --- |
| QMEAN | DisCo Global | 0.25 ± 0.09 |
| Clash Score | 4.68 |  |
| Ramachandran Favoured | 83.72% |  |
| Ramachandran Outliers | 3.49% | B326 VAL, B328 THR, B303 PRO |
| Rotamer Outliers | 1.56% | B319 TYR |
| C-Beta Deviations | 2 | B357 ASP, B326 VAL |
| Bad Bonds | 2 / 696 | B367 ASP-B368 ALA, B300 GLY |
| Bad Angles | 14 / 935 | B316 PHE, B356 GLN, B377 ASP, B387 PHE, B329 ASP, B319 TYR, B357 ASP, B313 ASP, B373 LYS, B338 LYS, (B338 LYS-B339 GLY), B328 THR, B326 VAL, B302 TYR |

Table S5. The toxicity prediction of the final epitopes in the vaccine structure. The results showed the non-toxicity of the vaccine structure

| Residues in red color represent the starting residue of toxic stretch for selected window length and those in blue color represent the trailing residues falling in toxic stretch. |
| --- |

| **Peptides Scanned from Original Protein** | | | | |  | | | | | | | | | | | | | | | | | | | | |
| --- | --- | --- | --- | --- | --- | --- | --- | --- | --- | --- | --- | --- | --- | --- | --- | --- | --- | --- | --- | --- | --- | --- | --- | --- | --- |
| [**Peptide Sequence**](https://webs.iiitd.edu.in/raghava/toxinpred/prot_submitfreq_S.php?ran=80427) | [**SVM score**](https://webs.iiitd.edu.in/raghava/toxinpred/prot_submitfreq_S.php?ran=80427) | [**Prediction**](https://webs.iiitd.edu.in/raghava/toxinpred/prot_submitfreq_S.php?ran=80427) | [**Hydrophobicity**](https://webs.iiitd.edu.in/raghava/toxinpred/prot_submitfreq_S.php?ran=80427) | [**Hydropathicity**](https://webs.iiitd.edu.in/raghava/toxinpred/prot_submitfreq_S.php?ran=80427) | [**Hydrophilicity**](https://webs.iiitd.edu.in/raghava/toxinpred/prot_submitfreq_S.php?ran=80427) | [**Charge**](https://webs.iiitd.edu.in/raghava/toxinpred/prot_submitfreq_S.php?ran=80427) | [**Mol wt**](https://webs.iiitd.edu.in/raghava/toxinpred/prot_submitfreq_S.php?ran=80427) |  |  |  |  |  |  |  |  |  |  |  |  |  |  |  |  |  |  |
| [MSSNVRDDSS](https://webs.iiitd.edu.in/raghava/toxinpred/pepsearch_S.php?seq=MSSNVRDDSS&thval=0.0) | -0.77 | Non-Toxin | -0.41 | -1.21 | 0.76 | -1.00 | 1097.24 |  |  |  |  |  |  |  |  |  |  |  |  |  |  |  |  |  |  |
| [SSNVRDDSSN](https://webs.iiitd.edu.in/raghava/toxinpred/pepsearch_S.php?seq=SSNVRDDSSN&thval=0.0) | -0.70 | Non-Toxin | -0.50 | -1.75 | 0.91 | -1.00 | 1080.15 |  |  |  |  |  |  |  |  |  |  |  |  |  |  |  |  |  |  |
| [SNVRDDSSNV](https://webs.iiitd.edu.in/raghava/toxinpred/pepsearch_S.php?seq=SNVRDDSSNV&thval=0.0) | -0.84 | Non-Toxin | -0.42 | -1.25 | 0.73 | -1.00 | 1092.21 |  |  |  |  |  |  |  |  |  |  |  |  |  |  |  |  |  |  |
| [NVRDDSSNVR](https://webs.iiitd.edu.in/raghava/toxinpred/pepsearch_S.php?seq=NVRDDSSNVR&thval=0.0) | -0.88 | Non-Toxin | -0.57 | -1.62 | 1.00 | 0.00 | 1161.32 |  |  |  |  |  |  |  |  |  |  |  |  |  |  |  |  |  |  |
| [VRDDSSNVRD](https://webs.iiitd.edu.in/raghava/toxinpred/pepsearch_S.php?seq=VRDDSSNVRD&thval=0.0) | -0.81 | Non-Toxin | -0.58 | -1.62 | 1.28 | -1.00 | 1162.30 |  |  |  |  |  |  |  |  |  |  |  |  |  |  |  |  |  |  |
| [RDDSSNVRDD](https://webs.iiitd.edu.in/raghava/toxinpred/pepsearch_S.php?seq=RDDSSNVRDD&thval=0.0) | -0.78 | Non-Toxin | -0.70 | -2.39 | 1.73 | -2.00 | 1178.25 |  |  |  |  |  |  |  |  |  |  |  |  |  |  |  |  |  |  |
| [DDSSNVRDDS](https://webs.iiitd.edu.in/raghava/toxinpred/pepsearch_S.php?seq=DDSSNVRDDS&thval=0.0) | -0.70 | Non-Toxin | -0.55 | -2.02 | 1.46 | -3.00 | 1109.14 |  |  |  |  |  |  |  |  |  |  |  |  |  |  |  |  |  |  |
| [DSSNVRDDSS](https://webs.iiitd.edu.in/raghava/toxinpred/pepsearch_S.php?seq=DSSNVRDDSS&thval=0.0) | -0.65 | Non-Toxin | -0.51 | -1.75 | 1.19 | -2.00 | 1081.13 |  |  |  |  |  |  |  |  |  |  |  |  |  |  |  |  |  |  |
| [SSNVRDDSSN](https://webs.iiitd.edu.in/raghava/toxinpred/pepsearch_S.php?seq=SSNVRDDSSN&thval=0.0) | -0.70 | Non-Toxin | -0.50 | -1.75 | 0.91 | -1.00 | 1080.15 |  |  |  |  |  |  |  |  |  |  |  |  |  |  |  |  |  |  |
| [SNVRDDSSNV](https://webs.iiitd.edu.in/raghava/toxinpred/pepsearch_S.php?seq=SNVRDDSSNV&thval=0.0) | -0.84 | Non-Toxin | -0.42 | -1.25 | 0.73 | -1.00 | 1092.21 |  |  |  |  |  |  |  |  |  |  |  |  |  |  |  |  |  |  |
| [NVRDDSSNVR](https://webs.iiitd.edu.in/raghava/toxinpred/pepsearch_S.php?seq=NVRDDSSNVR&thval=0.0) | -0.88 | Non-Toxin | -0.57 | -1.62 | 1.00 | 0.00 | 1161.32 |  |  |  |  |  |  |  |  |  |  |  |  |  |  |  |  |  |  |
| [VRDDSSNVRD](https://webs.iiitd.edu.in/raghava/toxinpred/pepsearch_S.php?seq=VRDDSSNVRD&thval=0.0) | -0.81 | Non-Toxin | -0.58 | -1.62 | 1.28 | -1.00 | 1162.30 |  |  |  |  |  |  |  |  |  |  |  |  |  |  |  |  |  |  |
| [RDDSSNVRDD](https://webs.iiitd.edu.in/raghava/toxinpred/pepsearch_S.php?seq=RDDSSNVRDD&thval=0.0) | -0.78 | Non-Toxin | -0.70 | -2.39 | 1.73 | -2.00 | 1178.25 |  |  |  |  |  |  |  |  |  |  |  |  |  |  |  |  |  |  |
| [DDSSNVRDDS](https://webs.iiitd.edu.in/raghava/toxinpred/pepsearch_S.php?seq=DDSSNVRDDS&thval=0.0) | -0.70 | Non-Toxin | -0.55 | -2.02 | 1.46 | -3.00 | 1109.14 |  |  |  |  |  |  |  |  |  |  |  |  |  |  |  |  |  |  |
| [DSSNVRDDSS](https://webs.iiitd.edu.in/raghava/toxinpred/pepsearch_S.php?seq=DSSNVRDDSS&thval=0.0) | -0.65 | Non-Toxin | -0.51 | -1.75 | 1.19 | -2.00 | 1081.13 |  |  |  |  |  |  |  |  |  |  |  |  |  |  |  |  |  |  |
| [SSNVRDDSSN](https://webs.iiitd.edu.in/raghava/toxinpred/pepsearch_S.php?seq=SSNVRDDSSN&thval=0.0) | -0.70 | Non-Toxin | -0.50 | -1.75 | 0.91 | -1.00 | 1080.15 |  |  |  |  |  |  |  |  |  |  |  |  |  |  |  |  |  |  |
| [SNVRDDSSNV](https://webs.iiitd.edu.in/raghava/toxinpred/pepsearch_S.php?seq=SNVRDDSSNV&thval=0.0) | -0.84 | Non-Toxin | -0.42 | -1.25 | 0.73 | -1.00 | 1092.21 |  |  |  |  |  |  |  |  |  |  |  |  |  |  |  |  |  |  |
| [NVRDDSSNVR](https://webs.iiitd.edu.in/raghava/toxinpred/pepsearch_S.php?seq=NVRDDSSNVR&thval=0.0) | -0.88 | Non-Toxin | -0.57 | -1.62 | 1.00 | 0.00 | 1161.32 |  |  |  |  |  |  |  |  |  |  |  |  |  |  |  |  |  |  |
| [VRDDSSNVRD](https://webs.iiitd.edu.in/raghava/toxinpred/pepsearch_S.php?seq=VRDDSSNVRD&thval=0.0) | -0.81 | Non-Toxin | -0.58 | -1.62 | 1.28 | -1.00 | 1162.30 |  |  |  |  |  |  |  |  |  |  |  |  |  |  |  |  |  |  |
| [RDDSSNVRDD](https://webs.iiitd.edu.in/raghava/toxinpred/pepsearch_S.php?seq=RDDSSNVRDD&thval=0.0) | -0.78 | Non-Toxin | -0.70 | -2.39 | 1.73 | -2.00 | 1178.25 |  |  |  |  |  |  |  |  |  |  |  |  |  |  |  |  |  |  |
| [DDSSNVRDDS](https://webs.iiitd.edu.in/raghava/toxinpred/pepsearch_S.php?seq=DDSSNVRDDS&thval=0.0) | -0.70 | Non-Toxin | -0.55 | -2.02 | 1.46 | -3.00 | 1109.14 |  |  |  |  |  |  |  |  |  |  |  |  |  |  |  |  |  |  |
| [DSSNVRDDSS](https://webs.iiitd.edu.in/raghava/toxinpred/pepsearch_S.php?seq=DSSNVRDDSS&thval=0.0) | -0.65 | Non-Toxin | -0.51 | -1.75 | 1.19 | -2.00 | 1081.13 |  |  |  |  |  |  |  |  |  |  |  |  |  |  |  |  |  |  |
| [SSNVRDDSSN](https://webs.iiitd.edu.in/raghava/toxinpred/pepsearch_S.php?seq=SSNVRDDSSN&thval=0.0) | -0.70 | Non-Toxin | -0.50 | -1.75 | 0.91 | -1.00 | 1080.15 |  |  |  |  |  |  |  |  |  |  |  |  |  |  |  |  |  |  |
| [SNVRDDSSNV](https://webs.iiitd.edu.in/raghava/toxinpred/pepsearch_S.php?seq=SNVRDDSSNV&thval=0.0) | -0.84 | Non-Toxin | -0.42 | -1.25 | 0.73 | -1.00 | 1092.21 |  |  |  |  |  |  |  |  |  |  |  |  |  |  |  |  |  |  |
| [NVRDDSSNVR](https://webs.iiitd.edu.in/raghava/toxinpred/pepsearch_S.php?seq=NVRDDSSNVR&thval=0.0) | -0.88 | Non-Toxin | -0.57 | -1.62 | 1.00 | 0.00 | 1161.32 |  |  |  |  |  |  |  |  |  |  |  |  |  |  |  |  |  |  |
| [VRDDSSNVRD](https://webs.iiitd.edu.in/raghava/toxinpred/pepsearch_S.php?seq=VRDDSSNVRD&thval=0.0) | -0.81 | Non-Toxin | -0.58 | -1.62 | 1.28 | -1.00 | 1162.30 |  |  |  |  |  |  |  |  |  |  |  |  |  |  |  |  |  |  |
| [RDDSSNVRDD](https://webs.iiitd.edu.in/raghava/toxinpred/pepsearch_S.php?seq=RDDSSNVRDD&thval=0.0) | -0.78 | Non-Toxin | -0.70 | -2.39 | 1.73 | -2.00 | 1178.25 |  |  |  |  |  |  |  |  |  |  |  |  |  |  |  |  |  |  |
| [DDSSNVRDDE](https://webs.iiitd.edu.in/raghava/toxinpred/pepsearch_S.php?seq=DDSSNVRDDE&thval=0.0) | -0.66 | Non-Toxin | -0.59 | -2.29 | 1.73 | -4.00 | 1151.18 |  |  |  |  |  |  |  |  |  |  |  |  |  |  |  |  |  |  |
| [DSSNVRDDEA](https://webs.iiitd.edu.in/raghava/toxinpred/pepsearch_S.php?seq=DSSNVRDDEA&thval=0.0) | -0.80 | Non-Toxin | -0.49 | -1.76 | 1.38 | -3.00 | 1107.17 |  |  |  |  |  |  |  |  |  |  |  |  |  |  |  |  |  |  |
| [SSNVRDDEAA](https://webs.iiitd.edu.in/raghava/toxinpred/pepsearch_S.php?seq=SSNVRDDEAA&thval=0.0) | -0.84 | Non-Toxin | -0.39 | -1.23 | 1.03 | -2.00 | 1063.16 |  |  |  |  |  |  |  |  |  |  |  |  |  |  |  |  |  |  |
| [SNVRDDEAAA](https://webs.iiitd.edu.in/raghava/toxinpred/pepsearch_S.php?seq=SNVRDDEAAA&thval=0.0) | -1.02 | Non-Toxin | -0.34 | -0.97 | 0.95 | -2.00 | 1047.16 |  |  |  |  |  |  |  |  |  |  |  |  |  |  |  |  |  |  |
| [NVRDDEAAAK](https://webs.iiitd.edu.in/raghava/toxinpred/pepsearch_S.php?seq=NVRDDEAAAK&thval=0.0) | -0.94 | Non-Toxin | -0.43 | -1.28 | 1.22 | -1.00 | 1088.26 |  |  |  |  |  |  |  |  |  |  |  |  |  |  |  |  |  |  |
| [VRDDEAAAKC](https://webs.iiitd.edu.in/raghava/toxinpred/pepsearch_S.php?seq=VRDDEAAAKC&thval=0.0) | -0.63 | Non-Toxin | -0.36 | -0.68 | 1.10 | -1.00 | 1077.29 |  |  |  |  |  |  |  |  |  |  |  |  |  |  |  |  |  |  |
| [RDDEAAAKCT](https://webs.iiitd.edu.in/raghava/toxinpred/pepsearch_S.php?seq=RDDEAAAKCT&thval=0.0) | -0.60 | Non-Toxin | -0.43 | -1.17 | 1.21 | -1.00 | 1079.26 |  |  |  |  |  |  |  |  |  |  |  |  |  |  |  |  |  |  |
| [DDEAAAKCTR](https://webs.iiitd.edu.in/raghava/toxinpred/pepsearch_S.php?seq=DDEAAAKCTR&thval=0.0) | -0.45 | Non-Toxin | -0.43 | -1.17 | 1.21 | -1.00 | 1079.26 |  |  |  |  |  |  |  |  |  |  |  |  |  |  |  |  |  |  |
| [DEAAAKCTRL](https://webs.iiitd.edu.in/raghava/toxinpred/pepsearch_S.php?seq=DEAAAKCTRL&thval=0.0) | -0.48 | Non-Toxin | -0.31 | -0.44 | 0.73 | 0.00 | 1077.34 |  |  |  |  |  |  |  |  |  |  |  |  |  |  |  |  |  |  |
| [EAAAKCTRLS](https://webs.iiitd.edu.in/raghava/toxinpred/pepsearch_S.php?seq=EAAAKCTRLS&thval=0.0) | -0.55 | Non-Toxin | -0.26 | -0.17 | 0.46 | 1.00 | 1049.33 |  |  |  |  |  |  |  |  |  |  |  |  |  |  |  |  |  |  |
| [AAAKCTRLSS](https://webs.iiitd.edu.in/raghava/toxinpred/pepsearch_S.php?seq=AAAKCTRLSS&thval=0.0) | -0.33 | Non-Toxin | -0.22 | 0.10 | 0.19 | 2.00 | 1007.29 |  |  |  |  |  |  |  |  |  |  |  |  |  |  |  |  |  |  |
| [AAKCTRLSSP](https://webs.iiitd.edu.in/raghava/toxinpred/pepsearch_S.php?seq=AAKCTRLSSP&thval=0.0) | 0.17 | Toxin | -0.26 | -0.24 | 0.24 | 2.00 | 1033.33 |  |  |  |  |  |  |  |  |  |  |  |  |  |  |  |  |  |  |
| [AKCTRLSSPT](https://webs.iiitd.edu.in/raghava/toxinpred/pepsearch_S.php?seq=AKCTRLSSPT&thval=0.0) | 0.08 | Toxin | -0.30 | -0.49 | 0.25 | 2.00 | 1063.36 |  |  |  |  |  |  |  |  |  |  |  |  |  |  |  |  |  |  |
| [KCTRLSSPTV](https://webs.iiitd.edu.in/raghava/toxinpred/pepsearch_S.php?seq=KCTRLSSPTV&thval=0.0) | -0.17 | Non-Toxin | -0.27 | -0.25 | 0.15 | 2.00 | 1091.42 |  |  |  |  |  |  |  |  |  |  |  |  |  |  |  |  |  |  |
| [CTRLSSPTVH](https://webs.iiitd.edu.in/raghava/toxinpred/pepsearch_S.php?seq=CTRLSSPTVH&thval=0.0) | -0.52 | Non-Toxin | -0.20 | -0.18 | -0.20 | 1.50 | 1100.39 |  |  |  |  |  |  |  |  |  |  |  |  |  |  |  |  |  |  |
| [TRLSSPTVHE](https://webs.iiitd.edu.in/raghava/toxinpred/pepsearch_S.php?seq=TRLSSPTVHE&thval=0.0) | -0.61 | Non-Toxin | -0.27 | -0.78 | 0.20 | 0.50 | 1126.37 |  |  |  |  |  |  |  |  |  |  |  |  |  |  |  |  |  |  |
| [RLSSPTVHEY](https://webs.iiitd.edu.in/raghava/toxinpred/pepsearch_S.php?seq=RLSSPTVHEY&thval=0.0) | -0.65 | Non-Toxin | -0.25 | -0.84 | 0.01 | 0.50 | 1188.44 |  |  |  |  |  |  |  |  |  |  |  |  |  |  |  |  |  |  |
| [LSSPTVHEYG](https://webs.iiitd.edu.in/raghava/toxinpred/pepsearch_S.php?seq=LSSPTVHEYG&thval=0.0) | -0.44 | Non-Toxin | -0.05 | -0.43 | -0.29 | -0.50 | 1089.31 |  |  |  |  |  |  |  |  |  |  |  |  |  |  |  |  |  |  |
| [SSPTVHEYGA](https://webs.iiitd.edu.in/raghava/toxinpred/pepsearch_S.php?seq=SSPTVHEYGA&thval=0.0) | -0.53 | Non-Toxin | -0.08 | -0.63 | -0.16 | -0.50 | 1047.22 |  |  |  |  |  |  |  |  |  |  |  |  |  |  |  |  |  |  |
| [SPTVHEYGAE](https://webs.iiitd.edu.in/raghava/toxinpred/pepsearch_S.php?seq=SPTVHEYGAE&thval=0.0) | -0.51 | Non-Toxin | -0.12 | -0.90 | 0.11 | -1.50 | 1089.26 |  |  |  |  |  |  |  |  |  |  |  |  |  |  |  |  |  |  |
| [PTVHEYGAEA](https://webs.iiitd.edu.in/raghava/toxinpred/pepsearch_S.php?seq=PTVHEYGAEA&thval=0.0) | -0.61 | Non-Toxin | -0.07 | -0.64 | 0.03 | -1.50 | 1073.26 |  |  |  |  |  |  |  |  |  |  |  |  |  |  |  |  |  |  |
| [TVHEYGAEAL](https://webs.iiitd.edu.in/raghava/toxinpred/pepsearch_S.php?seq=TVHEYGAEAL&thval=0.0) | -0.54 | Non-Toxin | -0.01 | -0.10 | -0.15 | -1.50 | 1089.31 |  |  |  |  |  |  |  |  |  |  |  |  |  |  |  |  |  |  |
| [VHEYGAEALE](https://webs.iiitd.edu.in/raghava/toxinpred/pepsearch_S.php?seq=VHEYGAEALE&thval=0.0) | -0.53 | Non-Toxin | -0.05 | -0.38 | 0.19 | -2.50 | 1117.32 |  |  |  |  |  |  |  |  |  |  |  |  |  |  |  |  |  |  |
| 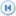 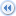  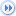 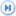                                  50                                  100                                 150                                 200                                 250                                 300                                 350                                 400 | | | | | | | | | | | | | | | | | | | | | | | | |  |
